# Targeted intracellular oral RNA delivery through tea polyphenol nanovesicle to outer membrane vesicle transfer for colitis treatment

**DOI:** 10.1126/sciadv.adx8336

**Published:** 2026-03-27

**Authors:** Taisong Fang, Songbai Liu

**Affiliations:** Department of Food Science and Nutrition, Zhejiang University, 866 Yuhangtang Road, Hangzhou 310058, China.

## Abstract

Efficient oral delivery of RNA to the target site is a long-standing issue for nucleic acid–based therapy. Herein, we adopted a vesicle-to-vesicle transfer strategy and established an efficient approach to encapsulate and stabilize RNA for targeted oral delivery. The amphiphilic specifically acylated epigallocatechin directly performed encapsulation of RNA and generated nanovesicles in high efficiency without assistance of additional materials. The RNA encapsulated in the nanovesicles was efficiently transferred to outer membrane vesicles (OMVs) derived from *Escherichia coli* Nissle 1917 probiotic through membrane fusion with simple operation. The derived hybrid vesicles (HVs) were further anchored with bilirubin and Lys-Asp-Glu-Leu grafted hyaluronic acid (HA-BR-KDEL) ligand for sequential cellular and intracellular targeting to the endoplasmic reticulum of inflammatory cells in inflamed intestinal tract. Oral delivery of HVs@HA-BR-KDEL notably alleviated colitis symptoms in mice and contributed to the restoration of intestinal homeostasis. The tea polyphenol hybrid OMV strategy holds great promise for oral gene-mediated treatment.

## Teaser

A vesicle-to-vesicle transfer strategy achieved efficient oral delivery of RNA to intracellular target site for colitis treatment.

## INTRODUCTION

Nucleic acid therapeutics, including small interfering RNA (siRNA), mRNA, and antisense oligonucleotides (ASOs), have recently emerged, greatly expanding the scope of candidate drugs and establishing themselves as a pivotal category of next-generation medicines (*1*–*4*). Oral delivery of RNA drugs is particularly attractive due to its convenience, fewer adverse effects, and high patient compliance (*5*). However, the ubiquitous presence of ribonucleases (RNases) and the complicated gastrointestinal (GI) environment pose great challenges for orally administered RNA drugs, leading to its degradation and compromised therapeutic efficacy (*6*). Therefore, oral RNA delivery techniques that prevent degradation and achieve targeted adhesion in the intestinal tract are required for enhancing treatment outcomes.

Tea polyphenols (TPs), the major bioactive components in tea, have excellent antioxidant activity. Extensive studies have shown that TPs can inhibit cell invasion and reduce the risk of cancer and degenerative diseases, garnering worldwide interest. Employment of TPs as carrier materials has unique advantages including oxidation resistance of the rich phenolic hydroxyl groups, polymerization properties, and broad binding and cross-linking capacity with nucleic acids and proteins, which can effectively improve the stability of the cargo molecules and exert synergistic biological effects (*7*–*9*). However, natural TPs have strong polarity and untamed assembly properties, and it is necessary to adjust their hydrophilicity for desired molecular assembly and improve their stability through molecular modification (*10*–*12*). Inspired by this concept, we reason that specifically modified amphiphilic TPs could directly encapsulate RNA and self-assemble into RNA-TP nanovesicles and without assistance of additional lipids.

Outer membrane vesicles (OMVs) secreted by Gram-negative bacteria are double-lipid layer nanostructures ranging from 20 to 400 nm in size (*13*–*15*), which can be steadily retained in the GI tract with low cytotoxicity and good biocompatibility (*16*–*18*). As a result, OMVs will be ideal carriers for oral administration of RNA. We speculate that the RNA-TP nanovesicles can be further fused with OMVs to form stable hybrid vesicles (HVs) that combine the benefits of both components. This strategy will effectively facilitate the encapsulation and stabilization of RNA for oral delivery. Inflammatory bowel disease (IBD), including Crohn’s disease (CD) and ulcerative colitis (UC), is a chronic GI disorder characterized by relapsing, uncontrolled and refractory inflammation (*19*, *20*). The endoplasmic reticulum (ER), which is responsible for protein folding, maturation, and trafficking, is intimately linked to inflammatory signaling pathways (*21*). To enhance IBD treatment efficacy, we designed a HA-BR-KDEL ligand for the cellular and intracellular sequential targeting of the ER in intestinal inflammatory cells. The ligand was constructed by grafting hyaluronic acid (HA; for CD44-mediated cellular targeting) with both the Lys-Asp-Glu-Leu (KDEL) peptide (for ER targeting) and bilirubin [BR; for its reactive oxygen species (ROS) scavenging and cytoprotective abilities] (*22*–*24*). The hydrophobic BR moiety allows for convenient anchoring of this ligand onto HVs, enabling specific sequential targeting.

In this study, epigallocatechin (EGC), a food-grade TP, was selectively acylated with palmitoyl chloride using the protection and deprotection approaches. Tumor necrosis factor–α siRNA (*TNF-*α siRNA), which silences the overexpression of this key inflammatory cytokine, was chosen as the therapeutic cargo (*25*). The amphiphilic EGC palmitate self-assembled and aggregated with siRNA through hydrogen bonding and hydrophobic interactions to form siRNA–EGC palmitate vesicles (sEPVs). Furthermore, sEPVs were efficiently incorporated with OMVs derived from the probiotic *Escherichia coli* Nissle 1917 (EcN) (*17*, *26*) to form HVs under sonication. Last, the HVs were anchored with HA-BR-KDEL. Subsequent in vitro and in vivo evaluations demonstrate that the orally administered HVs@HA-BR-KDEL platform exhibited outstanding efficacy against colitis, offering a convenient, safe, and effective strategy for oral siRNA delivery.

## RESULTS

### Specific acylation of EGC

Although epigallocatechin gallate (EGCG) is the major component of TPs, there are no appropriate hydroxyl groups for specific acylation according to our prior investigations (*27*). As the second major component, EGC has the essential aliphatic hydroxyl group on the C ring with little contribution to antioxidant activity, which can be selectively acylated without perturbation of phenolic hydroxyl groups (*10*). The interactions between polyphenols and nucleic acids rely primarily on hydrogen bonding and hydrophobic interactions, both mediated by phenolic hydroxyl and aromatic groups (*28*). Therefore, specific acylation of EGC with hydrophobic long-chain fatty acids can confer amphiphilic and self-assembly properties while maximally preserving its inherent antioxidant activity and RNA binding capacity (*11*, *12*).

**Fig. 1.**
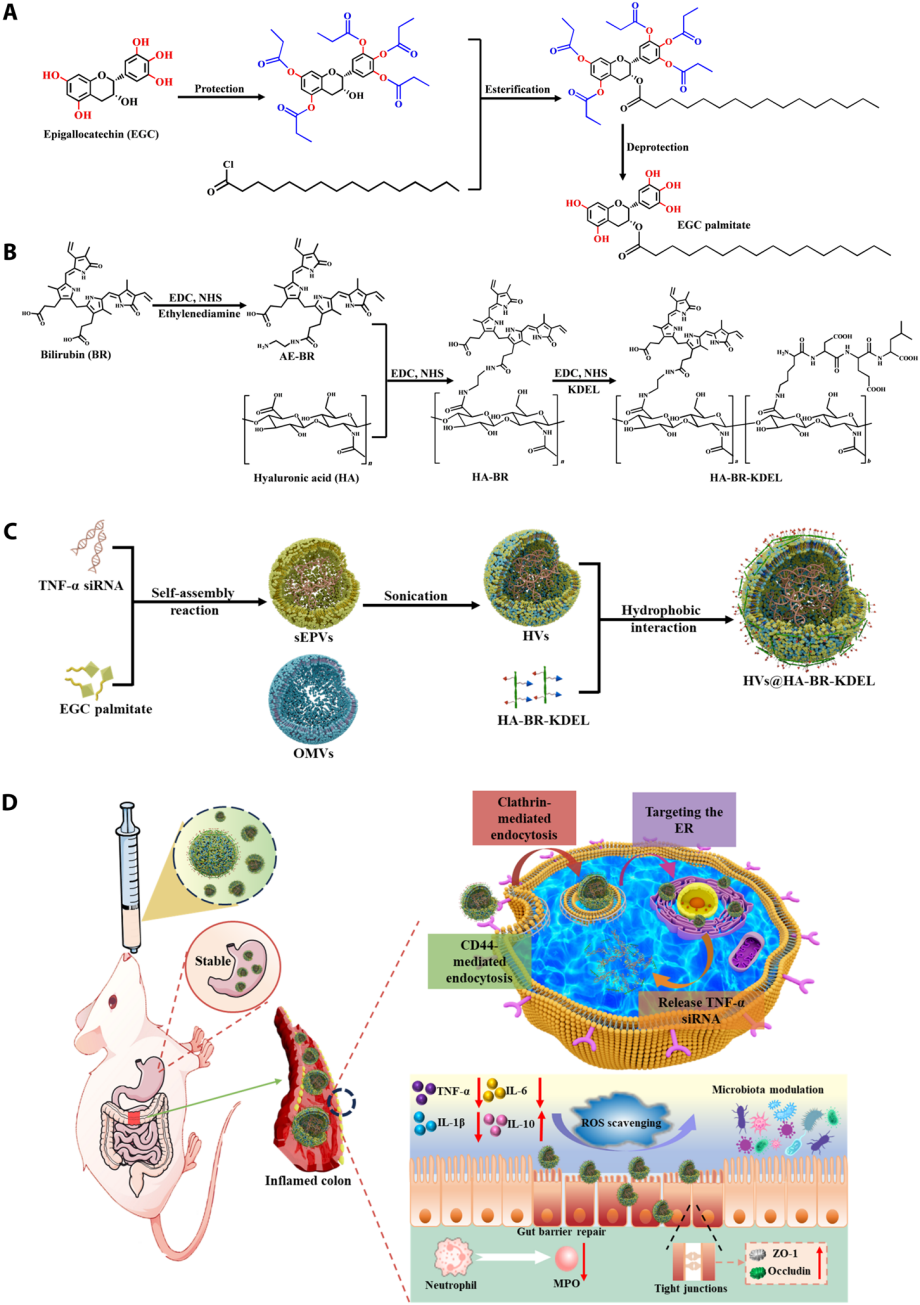
Schematic illustration for the preparation of HVs@HA-BR-KDEL and applications in the treatment of DSS-induced colitis in mice. (**A** and **B**) Synthetic scheme of (A) EGC palmitate and (B) HA-BR-KDEL. (**C**) Preparation procedure of HVs@HA-BR-KDEL. (**D**) Schematic illustrating the underlying targeting mechanism of HVs@HA-BR-KDEL toward inflammatory cells and its therapeutic efficacy in a DSS-induced colitis mouse model.

The frequently used palmitic acid for acylation of polyphenols was applied for specific acylation of EGC. Using selective protection and deprotection strategies developed by our group, the target compound (EGC palmitate) was prepared in a high overall yield of 89%. The overall synthetic route is outlined in Fig. 1A. Thin-layer chromatography (TLC) analysis revealed the smooth production and complete separation of EGC palmitate from the starting materials and intermediates (fig. S1A). The structure and purity of the product were confirmed by nuclear magnetic resonance (NMR) and high-performance liquid chromatography–mass spectrometry (HPLC-MS) analysis. Compared to natural EGC, the ^1^H NMR spectrum of EGC palmitate displayed prominent new signals around 0.8 to 1.4 parts per million (ppm), confirming the successful incorporation of the palmitoyl chain (fig. S1B). HPLC-MS analysis showed a single, well-separated peak corresponding to EGC palmitate with high purity (95%). Its structural identity was further verified by electrospray ionization mass spectrometry (ESI-MS) spectrum in negative ion mode, which exhibited a characteristic ion at mass/charge ratio (*m/z*) 1087.18 (fig. S1, C and D). Collectively, these data confirm the successful synthesis of specifically acylated EGC palmitate.

### Preparation and characterization of HVs

**Fig. 2.**
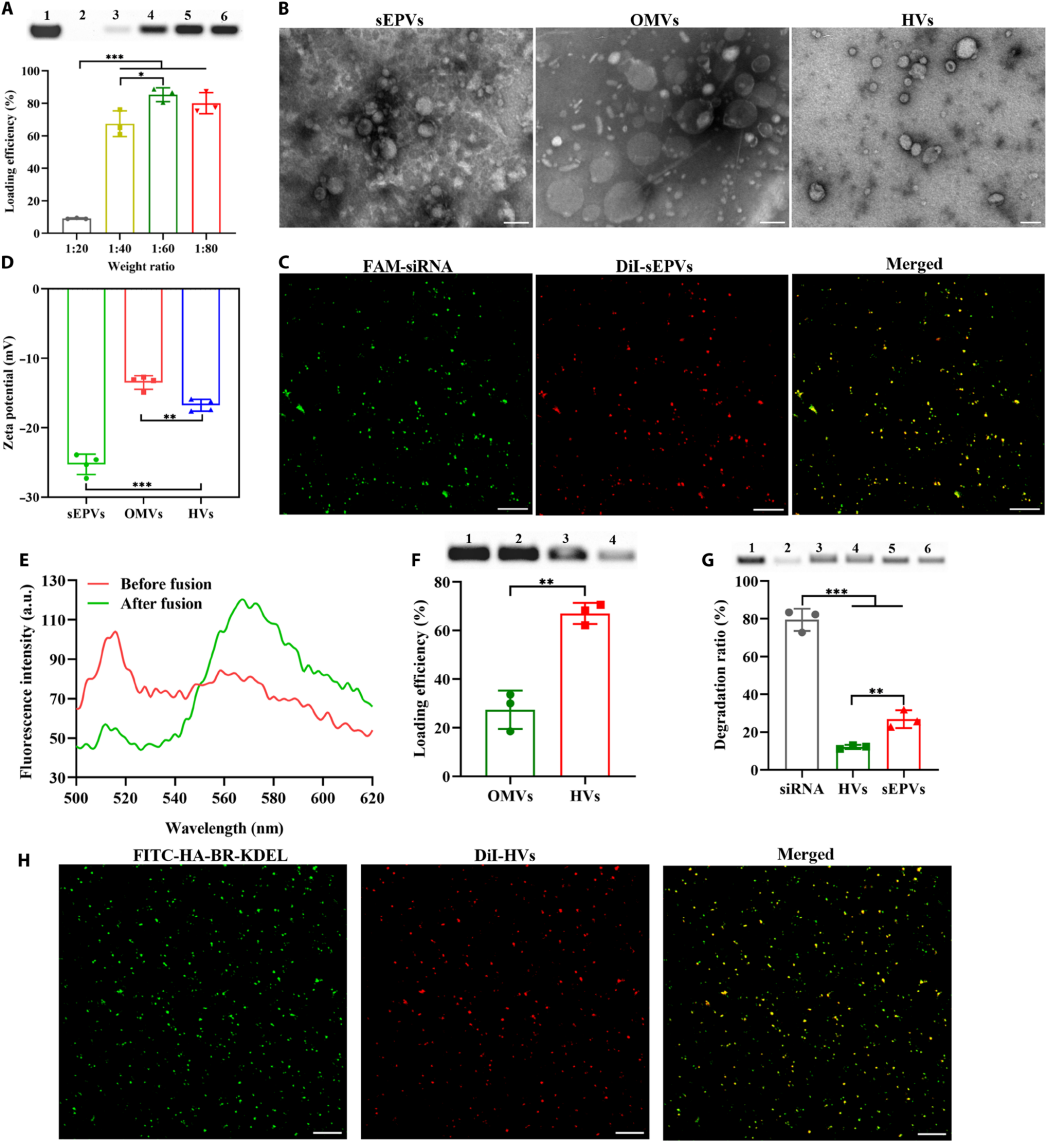
Characterization of nanovesicles. (**A**) siRNA loading efficiency of sEPVs at different siRNA–to–EGC palmitate weight ratios, as determined by gel electrophoresis (lanes 1 and 2: permeate and retentate of naked siRNA; lanes 3 to 6: retentate of sEPVs at weight ratios of 1:20, 1:40, 1:60, and 1:80, respectively) and quantitative analysis (*n* = 3). (**B**) Representative TEM images of sEPVs, OMVs, and HVs. Scale bars, 100 nm. (**C**) CLSM images of dye-labeled sEPVs, with siRNA labeled with FAM (green) and the sEPV membrane labeled with DiI (red). Scale bars, 5 μm. (**D**) Surface zeta potential of sEPVs, OMVs, and HVs. (**E**) Emission spectra from a FRET assay, with sEPVs labeled with DiO and OMVs labeled with DiI. a.u., arbitrary units. (**F**) siRNA loading efficiency of OMVs and HVs, shown by gel electrophoresis (lane 1: naked siRNA before ultrafiltration; lane 2: permeate of naked siRNA; lane 3: permeate of siRNA-OMVs; lane 4: permeate of HVs) and quantitative analysis (*n* = 3). (**G**) Protection of siRNA against RNase degradation by nanovesicles. Gel images show samples before and after RNase treatment (lanes 1 and 2: naked siRNA; lanes 3 and 4: HVs; lanes 5 and 6: sEPVs), with quantitative analysis of siRNA degradation ratios. (**H**) CLSM images of HVs@HA-BR-KDEL, showing the anchoring of FITC-labeled HA-BR-KDEL (green) onto DiI-labeled HVs (red). Scale bars, 5 μm. Data are presented as the means ± SD. *P < 0.05; **P < 0.01; ***P < 0.001.

TNF-α, a key inflammatory cytokine that can result in an imbalanced colonic microenvironment in IBD, was selected as the therapeutic target (*29*). As a proof-of-concept demonstration, we designed a *TNF-*α siRNA and evaluated its delivery potential using EGC palmitate as a vector. It was revealed that mixing EGC palmitate with *TNF-*α siRNA successfully generated nanovesicles via self-assembly reaction without assistance of other lipids that are generally required, which considerably facilitates RNA encapsulation. We propose a mechanism whereby the phenolic hydroxyl groups of EGC palmitate direct hydrogen bonding and hydrophobic interactions with siRNA and perform encapsulation of siRNA in the core. Simultaneously, the hydrophobic fatty acid chains of EGC palmitate self-assemble into an outer vesicular layer to form nanovesicles. The loading efficiency of sEPVs was quantified by agarose gel electrophoresis (*9*, *30*). As shown in Fig. 2A, the optimal loading efficiency reached 85% at a weight ratio (siRNA:EGC palmitate) of 1:60, compared to other tested ratios (1:20, 1:40, and 1:80), demonstrating the excellent loading capacity of sEPVs. On the basis of this result, the 1:60 ratio was selected for follow-up experiments. To characterize the sEPV structure, we used multiple techniques. Transmission electron microscopy (TEM) images showed that the sEPVs had a well-defined spherical morphology (Fig. 2B). Besides, the size distribution was determined by nanoparticle tracking analysis (NTA), giving an average size of ~156 nm with a narrow size distribution (fig. S2A). The smaller particle sizes observed by TEM, compared to NTA, are attributed to the differences in measurement principles. Specifically, TEM reports the dry core diameter under vacuum, whereas NTA measures the hydrodynamic diameter, which refers to the size of the hydrated vesicle including its solvation layer. To track the nanovesicles, siRNA was labeled with 5′-fluorescein phosphoramidite (FAM; green fluorescence) and sEPVs were labeled with 1,1′-dioctadecyl-3,3,3′,3′-tetramethylindocarbocyanine perchlorate (DiI; red fluorescence). Confocal laser scanning microscopy (CLSM) images revealed marked colocalization of the green and red fluorescent signals, confirming the encapsulation of siRNA inside the sEPVs (Fig. 2C).

OMVs were isolated from the probiotic EcN using a well-established ultracentrifugation method (*31*). Characterization by TEM and NTA showed that the purified OMVs had a spherical morphology with an average size of ~175 nm (Fig. 2B and fig. S2B), similar to that of sEPVs. Subsequently, HVs were constructed by fusing sEPVs and OMVs via sonication. TEM imaging revealed that the HVs maintained a similar spherical morphology, making them morphologically indistinguishable from the parent vesicles (Fig. 2B). However, NTA measurements indicated a significant increase in the average size of HVs (194 nm) compared to individual sEPVs or OMVs (fig. S2C). Consistent with a prior study (*32*), the surface zeta potential of HVs, as measured by dynamic light scattering (DLS), was significantly decreased relative to OMVs (*P* < 0.01) (Fig. 2D), a change attributable to the incorporation of the negatively charged sEPVs.

The membrane fusion was confirmed by CLSM examination as described previously (*33*). As shown in fig. S3, the prepared HVs exhibited extensive colocalization (yellow) of FAM-siRNA (green) from sEPVs and DiI (red) from OMVs. In contrast, the nonsonicated mixture of sEPVs and OMVs displayed distinct and separate green and red fluorescence signals. This clear visual contrast confirms that sonication induces membrane fusion, leading to the successful formation of HVs and the loading of FAM-siRNA. To further quantify fusion efficiency, we performed particle-based colocalization analysis on the confocal images using the ImageJ software. Individual nanovesicles were categorized as “green-only” (FAM-sEPVs), “red-only” (DiI-OMVs), or “yellow” (colocalized, indicating fused HVs). The analysis revealed that the apparent fusion efficiency, defined as the percentage of colocalized particles, reached 56.8% in the sonication-fused HV group. In contrast, not a single colocalized particle was observed in the simple physical mixture group, resulting in a fusion efficiency of 0%. These results quantitatively demonstrate the high efficiency of our sonication-mediated fusion approach. In addition, the membrane fusion was confirmed by Förster resonance energy transfer (FRET) using the fluorophore pair 3,3′-dioctadecyloxacarbocyanine perchlorate (DiO) and DiI, a well-established method for monitoring membrane fusion (*34*, *35*). Successful fusion between DiI-labeled OMVs and DiO-labeled sEPVs brings the fluorophores into close proximity, leading to an increase in FRET efficiency. This was evidenced by a marked change in the emission spectra (Fig. 2E). Before fusion, the spectrum was dominated by a strong DiO emission peak at 510 nm. After fusion, a strong DiI emission peak at 565 nm emerged, demonstrating the occurrence of FRET. These results collectively verify the successful formation of HVs through the membrane fusion of sEPVs and OMVs.

Last, the siRNA loading efficiency of the HVs or OMVs was quantified using ultrafiltration combined with agarose gel electrophoresis (Fig. 2F). The analysis revealed that 67% of the siRNA was successfully encapsulated within the HVs. In contrast, when siRNA was directly loaded into OMVs via sonication without prior encapsulation in sEPVs, the loading efficiency dropped markedly to only 27% (*P* < 0.01). The notably decreased loading capacity highlights the superior efficiency of sEPVs for transferring RNA into OMVs via membrane fusion, which is intriguing for efficient RNA delivery.

### Stability of siRNA in nanovesicles in vitro

To assess RNase resistance, HVs, sEPVs, or naked siRNA was treated with RNase (2 ng/μl) for 5 min before the reaction was terminated with an RNase inhibitor. Electrophoretic analysis (Fig. 2G) revealed that naked siRNA, lacking any protective encapsulation, was completely degraded. In contrast, both HVs and sEPVs efficiently prevented siRNA from enzymatic degradation, preserving 88 and 73% of the siRNA, respectively. We further evaluated the stability of the nanovesicles in simulated gastric fluid (SGF) at 37°C for 2 hours. As shown in fig. S4, the OMV membrane provided effective protection for encapsulated siRNA, resulting in significantly greater stability in HVs than in sEPVs. Consequently, the degradation ratio was much lower for HVs (58%) than for sEPVs (97%). Collectively, these results confirm that the HVs provide considerable stability and effectively protect encapsulated siRNA from harsh GI environments.

### Preparation of HVs@HA-BR-KDEL

BR, a hydrophobic by-product of hemoglobin breakdown found in bile, has strong antioxidant and cytoprotective properties (*36*, *37*). HA is a biocompatible and biodegradable polysaccharide that can effectively target CD44, which is overexpressed on inflammatory cells (*38*). Therefore, we grafted BR onto the HA backbone. This conjugation provides lipophilic side chains for insertion into the HVs membrane and protects the HA from hyaluronidase-mediated degradation in the inflamed colon. Furthermore, accumulating evidence reveals that ER stress is intimately involved in the pathogenesis of IBD, regulation of which is considered as an effective therapeutic approach (*39*–*41*). Inspired by the superior ER-targeting capability of the KDEL peptide, we grafted it onto the HA backbone. The successful synthesis of the HA-BR-KDEL conjugate was confirmed by ^1^H NMR spectroscopy (fig. S5), which exhibited characteristic signals from HA (3.1 to 3.8 ppm; sugar ring protons), BR (2.1 to 2.2 ppm; pyrrole aliphatic side chain), and KDEL (0.7 to 0.8 ppm; leucine side chain). The conjugate was introduced to HVs, followed by sonication and subsequent incorporation into the membrane via magnetic stirring for 30 min at room temperature (RT), yielding the final HVs@HA-BR-KDEL. CLSM analysis confirmed the successful modification, as evidenced by the prominent colocalization (yellow) of the blue fluorescence from fluorescein isothiocyanate (FITC)–labeled HA-BR-KDEL and the red fluorescence from DiI-labeled HVs (Fig. 2H), demonstrating the effective insertion of the conjugate into the HVs membrane.

### Cellular uptake of HVs@HA-BR-KDEL

The RAW264.7 murine macrophage cell line, which has high phagocytic capacity and is a well-established model frequently used to study cellular uptake and inflammatory responses in vitro (*8*, *30*, *42*), was used in our research. To verify that HA-CD44 binding mediates the endocytosis of HVs@HA-BR-KDEL, we first assessed CD44 overexpression on the lipopolysaccharide (LPS)–stimulated inflammatory RAW264.7 cells via flow cytometry using an FITC-conjugated anti-CD44 antibody (BD Biosciences, USA). As shown in fig. S6, the quantitative results exhibited that CD44 expression was significantly up-regulated on the inflammatory RAW264.7 cells compared to the normal control cells (3.4-fold increase, *P* < 0.001). These results not only confirm the overexpression of CD44 on the inflammatory RAW264.7 cells but also strengthen our speculation that CD44 receptor plays a crucial role in mediating the HVs@HA-BR-KDEL uptake.

**Fig. 3.**
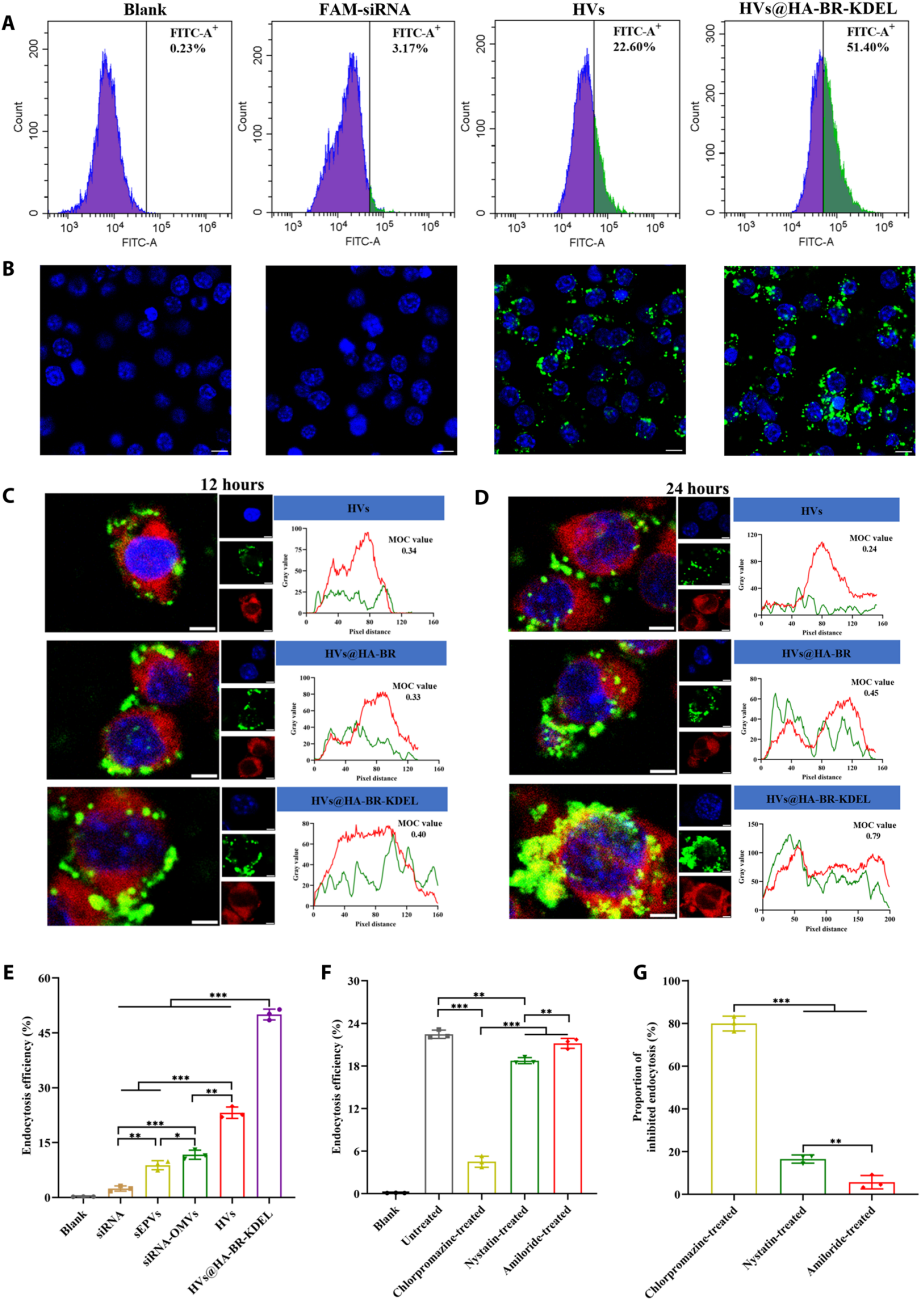
Cellular uptake in vitro. (**A**) Flow cytometric analysis and (**B**) CLSM images of inflammatory RAW264.7 cells treated with naked or nanovesicle-encapsulated FAM-siRNA for 24 hours. Green: FAM-siRNA; blue: Hoechst 33342 (nuclei). Scale bars, 10 μm. (**C** and **D**) CLSM images showing the subcellular localization of HVs@HA-BR-KDEL (green; FAM-siRNA) after (C) 12 hours and (D) 24 hours of incubation. The ER is stained in red (Tracker Red), and nuclei are stained in blue (Hoechst 33342). Scale bars, 5 μm. (**E**) Quantitative analysis of the cellular uptake efficiency of naked FAM-siRNA and FAM-siRNA delivered by sEPVs, OMVs, HVs, or HVs@HA-BR-KDEL after 24 hours of incubation (*n* = 3). (**F**) Quantitative analysis of the cellular internalization of HVs@HA-BR-KDEL after 12 hours of incubation following pretreatment with various endocytosis inhibitors (*n* = 3). (**G**) Contribution of different endocytic pathways to the cellular internalization of HVs@HA-BR-KDEL (*n* = 3). Data are presented as the means ± SD. *P < 0.05; **P < 0.01; ***P < 0.001.

Subsequently, cellular uptake of HVs@HA-BR-KDEL loading FAM-siRNA by inflammatory RAW264.7 cells was evaluated. As expected, quantitative analysis of endocytosis efficiency in inflammatory RAW264.7 cells by flow cytometry disclosed that the green fluorescence intensity of cells treated with HVs@HA-BR-KDEL was significantly higher than that of the naked siRNA (16.2-fold increase, *P* < 0.001) or HV group (2.3-fold increase, *P* < 0.001) (Fig. 3, A and E). Furthermore, CLSM images (Fig. 3B) demonstrated that no green fluorescence observed in the naked siRNA group after incubation for 24 hours, indicating that naked siRNA hardly entered RAW264.7 cells. In contrast, prominent green fluorescence was observed upon administering of HVs and HVs@HA-BR-KDEL, which certified endocytosis of these nanovesicles by RAW264.7 cells. In particular, after anchoring the HA-BR-KDEL to the membrane of HVs, the cellular green fluorescence was remarkably increased. Similar results were also observed at 6 and 12 hours (fig. S7). A comparative analysis of endocytosis efficiency among sEPVs, siRNA-OMVs, HVs, and HVs@HA-BR-KDEL after 24-hour incubation was also conducted. As shown in Fig. 3E and fig. S8, the siRNA-OMV group exhibited higher endocytosis efficiency than the sEPV group (1.5-fold, *P* < 0.05). The intrinsic immunogenicity of OMVs, derived from parent bacterial outer membrane and periplasmic components, promotes their efficient phagocytosis by RAW264.7 macrophages, surpassing the uptake efficiency of synthetic sEPVs (*43*, *44*). Notably, the HVs exhibited a significant improvement in endocytosis efficiency over both the sEPVs (2.6-fold, *P* < 0.001) and siRNA-OMVs (2.0-fold, *P* < 0.01) (Fig. 3E). This enhancement is likely attributable to the synergistic design of HVs, which combines the intrinsic stability of the siRNA–EGC palmitate complex with the inherent biological activity of the OMV membrane, the latter potentially engaging natural endocytic pathways. However, HVs@HA-BR-KDEL achieved the highest endocytosis efficiency, suggesting that the HA-CD44 targeting pathway is a more dominant factor for cellular uptake than the intrinsic properties of the original nanovesicles. To further confirm the role of the HA-CD44 targeting pathway, cells were pretreated with an anti-CD44 antibody for 2 hours at 4°C before the addition of DiI-labeled HVs@HA-BR-KDEL. After 24 hours of treatment, antibody pretreatment significantly reduced endocytosis efficiency of HVs@HA-BR-KDEL by 40.7% (*P* < 0.001) compared with treatment by HVs@HA-BR-KDEL alone, as measured through cellular red fluorescence intensity (fig. S9). It is worth noting that a difference existed between the endocytosis efficiencies calculated on the basis of DiI-nanovesicles (76.5%) and FAM-siRNA (51.4%). This discrepancy suggests that a fraction of siRNA may be degraded within lysosomes. Overall, these results demonstrate that HA-functionalized HVs achieved their efficient binding to the CD44 receptors overexpressed by inflammatory RAW264.7 cells, ultimately resulting in markedly enhanced endocytosis efficiency.

The ER targeting capability of HVs@HA-BR-KDEL was further evaluated using an organelle colocalization assay. Inflammatory RAW264.7 cells were treated with HVs, HVs@HA-BR, or HVs@HA-BR-KDEL for 12 to 24 hours, followed by staining with ER-Tracker Red (for the ER) and Hoechst 33342 (for nuclei). Using Manders overlap coefficient (MOC) to evaluate colocalization, we applied the generally accepted criteria that values greater than 0.6 indicate a strong colocalization, whereas values below 0.4 suggest no colocalization (*45*, *46*). Confocal images revealed a high MOC value in the HVs@HA-BR-KDEL group after 24-hour incubation (MOC = 0.79; Fig. 3D), which was markedly greater than the values observed for the other two control groups: HVs (MOC = 0.24) and HVs@HA-BR (MOC = 0.45). These results demonstrate the active homing of the nanovesicles to the ER, which resulted in the precise localization of FAM-siRNA within this organelle. However, a weaker fluorescence overlap observed at 12 hours suggests that efficient siRNA delivery to the ER requires prolonged incubation time (Fig. 3C). These results verify that the KDEL-grafted HA is essential for achieving sequential targeting to the ER of inflammatory cells.

To elucidate the cellular uptake pathway of HVs@HA-BR-KDEL, we used a pharmacological inhibition assay using chlorpromazine (inhibitor of clathrin-mediated endocytosis), nystatin (inhibitor of caveolin-mediated endocytosis), and amiloride (inhibitor of macropinocytosis) (*47*). RAW264.7 cells were pretreated with each inhibitor before incubation with HVs@HA-BR-KDEL for 12 hours. Flow cytometry analysis revealed HVs@HA-BR-KDEL internalization in the presence or absence of endocytosis inhibitors, as indicated by green fluorescence shifts of FAM-siRNA (fig. S10). For these endocytosis inhibitors, chlorpromazine elicited the most pronounced inhibition of HVs@HA-BR-KDEL internalization with the lowest endocytosis efficiency of 5.3% (Fig. 3F), suggesting a primary role for clathrin-mediated endocytosis. Quantitative assessment of the inhibition profiles enabled estimation of the relative contribution of each pathway, indicating that HVs@HA-BR-KDEL enters cells predominantly via clathrin-mediated endocytosis (79.9%), with caveolin-mediated endocytosis (16.5%) and macropinocytosis (5.7%) playing minor roles (Fig. 3G). These findings were confirmed by confocal images, which showed a substantial reduction in fluorescence signal upon chlorpromazine treatment, further underscoring the critical dependence on clathrin-mediated uptake (fig. S11).

### ROS scavenging and anti-inflammatory activity in vitro

Infiltrated macrophages are pivotal effector cells in IBD pathogenesis. As a major source of pro-inflammatory cytokines [including TNF-α, interleukin-6 (IL-6), and interleukin-1β (IL-1β)], they drive tissue damage, making them a core therapeutic target (*48*). Because LPS-stimulated RAW264.7 macrophages highly express CD44 and mimic the activated phenotype found in colitis, they provide a relevant in vitro model for evaluating the CD44-mediated targeting, antioxidant, and anti-inflammatory efficacy of our engineered nanovesicles.

**Fig. 4.**
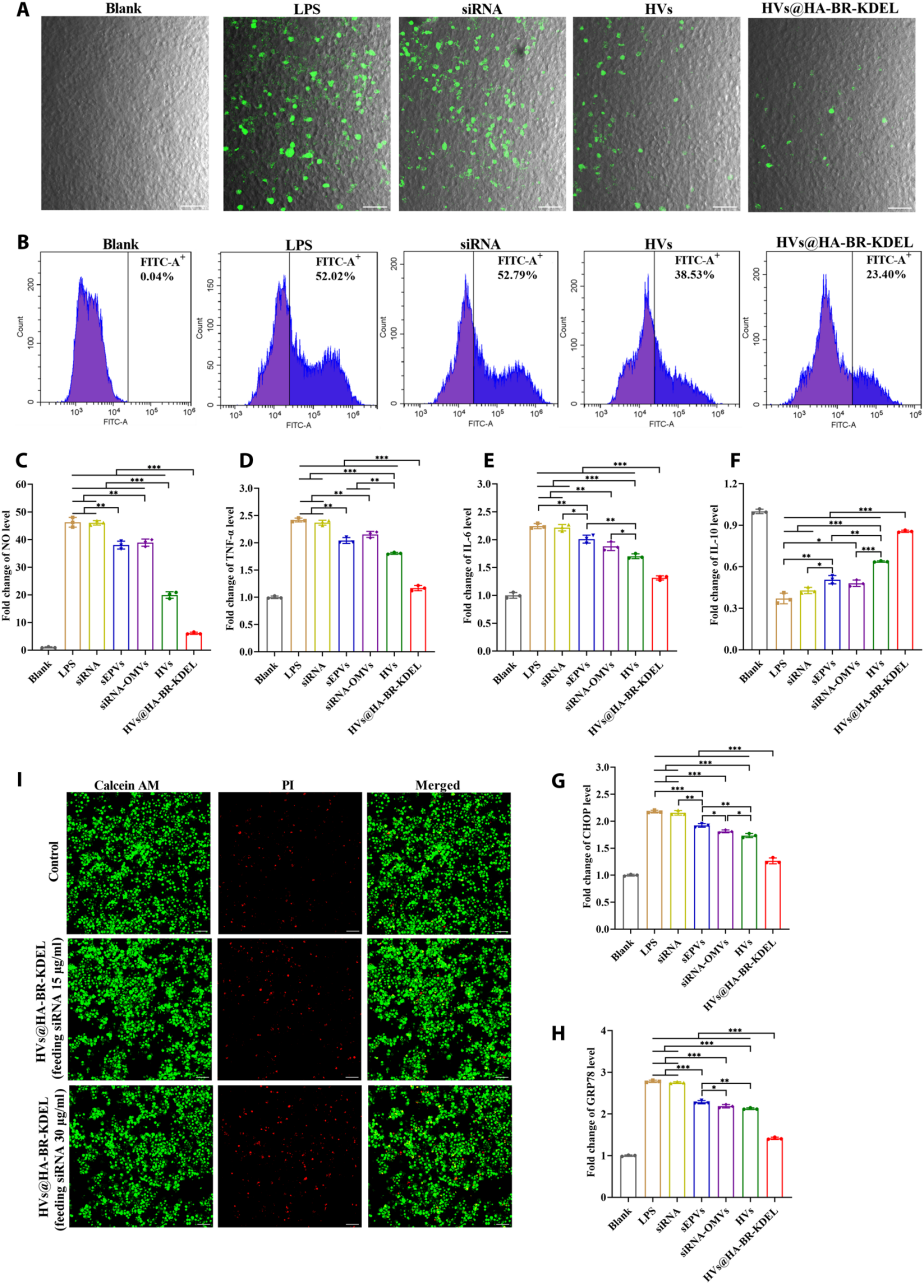
ROS scavenging and anti-inflammatory activity in vitro. (**A**) Fluorescence images of DCFH-DA–labeled RAW264.7 cells showing intracellular ROS production. Scale bars, 50 μm. (**B**) Flow cytometric analysis of ROS levels in DCFH-DA–labeled RAW264.7 cells. (**C** to **F**) Relative levels of (C) NO (measured as nitrite), (D) TNF-α, (E) IL-6, and (F) IL-10 in cell culture media after different treatments (*n* = 3). (**G** and **H**) Protein expression levels of (G) CHOP and (H) GRP78 in RAW264.7 cells, as determined by ELISA (*n* = 3). (**I**) Live/dead staining of RAW264.7 cells after different treatments. Scale bars, 50 μm. Data are presented as the means ± SD. *P < 0.05; **P < 0.01; ***P < 0.001.

The protective effect of HVs@HA-BR-KDEL against ROS-induced damage was evaluated using an LPS-induced inflammatory model in RAW264.7 cells. Intracellular ROS levels were measured by flow cytometry and CLSM using a 2′,7′-dichlorofluorescein diacetate (DCFH-DA) probe following treatment with the nanovesicles. As shown in Fig. 4 (A and B) and fig. S12, the LPS group exhibited the highest ROS fluorescence intensity (52.02%), which was not reduced by naked siRNA treatment (52.79%). In contrast, all nanovesicle-based siRNA delivery systems significantly attenuated intracellular ROS levels. Notably, sEPVs demonstrated superior antioxidant effects over siRNA-OMVs (*P* < 0.05), likely due to their high EGC palmitate payload. This molecule serves as a source of abundant phenolic groups for direct ROS scavenging, whereas siRNA-OMVs rely on less efficient bacterial membrane components (*49*, *50*). The HV group showed stronger antioxidant activity than siRNA-OMVs (*P* < 0.05) but similar to sEPVs, suggesting a synergistic integration of EGC palmitate’s ROS scavenging ability with residual biological activity of OMVs. HVs@HA-BR-KDEL showed the highest ROS scavenging efficacy among all groups (*P* < 0.001), owing to its enhanced CD44-mediated uptake, KDEL-directed ER delivery for intracellular action, and the inherent antioxidant capacity of EGC palmitate and BR, which collectively confer superior protection against oxidative stress.

Moreover, the anti-inflammatory capacity of HVs@HA-BR-KDEL was evaluated by monitoring the expression of key cytokines in LPS-stimulated RAW264.7 macrophages. Nitric oxide (NO), which is overproduced during cell inflammatory responses and contributes to tissue damage (*51*), was significantly suppressed by HVs@HA-BR-KDEL treatment compared to other groups (*P* < 0.001) (Fig. 4C). Furthermore, HVs@HA-BR-KDEL markedly inhibited the secretion of pro-inflammatory cytokines, including TNF-α and IL-6 (Fig. 4, D and E), while elevating the level of the anti-inflammatory cytokine IL-10 (Fig. 4F). In addition, HVs demonstrated superior anti-inflammatory efficacy over both sEPVs and siRNA-OMVs (which were comparable), due to a synergistic effect from combining OMV membranes with sEPVs, which enhanced modulation of inflammatory responses. Among all groups, HVs@HA-BR-KDEL demonstrated the strongest anti-inflammatory activity, which can be attributed to its more efficient cellular internalization and additional anti-inflammatory effect of the membrane-anchored BR, enabling comprehensive suppression of inflammation.

To investigate whether the anti-inflammatory effect of HVs@HA-BR-KDEL was associated with the modulation of ER stress, we quantified the protein levels of two representative ER stress markers, C/EBP homologous protein (CHOP), and glucose-regulated protein 78 (GRP78), using enzyme-linked immunosorbent assay (ELISA). As shown in Fig. 4 (G and H), siRNA-OMVs more effectively suppressed ER stress compared to sEPVs (*P* < 0.05), which may be attributed to the inherent biological activity of bacterial outer membrane components that modulate cellular stress responses. A synergy between OMVs and sEPVs underlies the superior efficacy of HVs in suppressing CHOP levels compared to the individual components. Although HVs reduced GRP78 more effectively than sEPVs, they showed no advantage over siRNA-OMVs. This differential effect may be explained by the distinct roles of the two markers. CHOP is more directly associated with apoptosis induced by severe ER stress and may be particularly sensitive to the combined properties of HVs, whereas GRP78, as a central ER chaperone, is predominantly regulated by OMV components, thereby reducing the additional benefit that could be offered by sEPVs in the hybrid system. In contrast, HVs@HA-BR-KDEL exhibited the most significant inhibition of the two ER stress markers among all groups (*P* < 0.001), which can be attributed to a synergy between enhanced ER-targeted siRNA delivery and anti-inflammatory actions of components. In summary, these results indicate that the alleviation of LPS-induced cellular inflammation by HVs@HA-BR-KDEL was associated with the suppression of ER stress.

Last, the biosafety of HVs@HA-BR-KDEL was evaluated. The Cell Counting Kit-8 (CCK-8) assay showed no cytotoxicity at the initial feeding siRNA concentration range of 5 to 30 μg/ml, with insignificant differences in cell viability compared to the control group (fig. S13). This was further confirmed by live/dead cell staining (Fig. 4I). Consequently, HVs@HA-BR-KDEL is a safe vector for siRNA delivery.

### Safety and biodistribution of HVs@HA-BR-KDEL in vivo

**Fig. 5.**
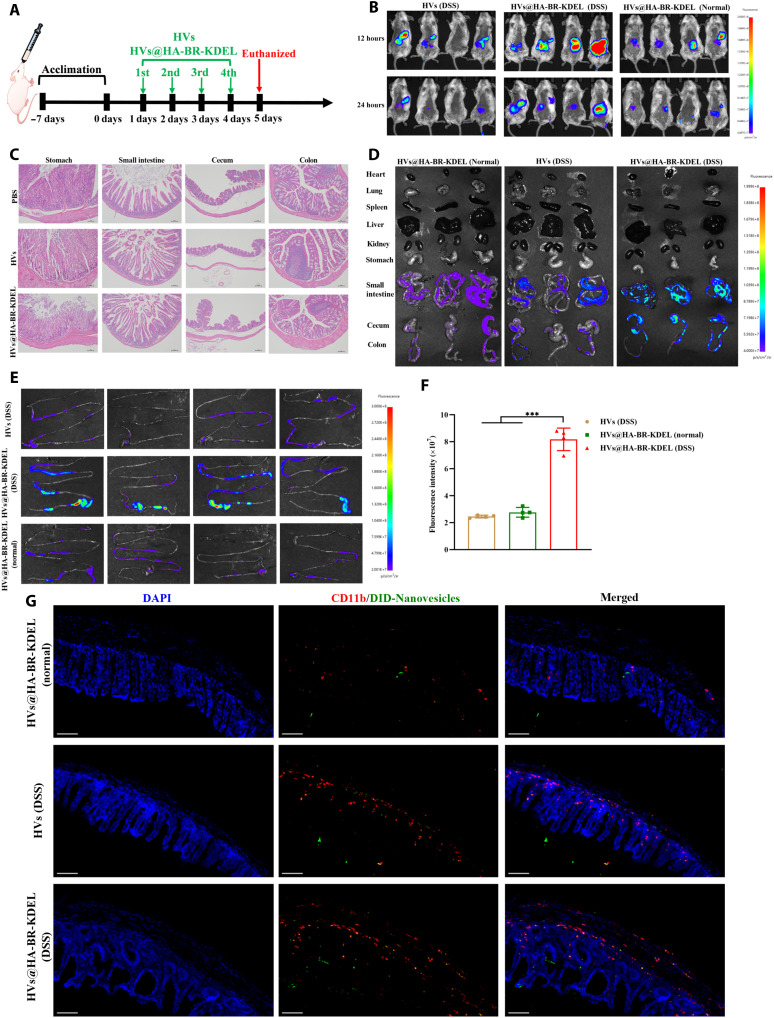
In vivo biosafety and biodistribution of HVs@HA-BR-KDEL. (**A**) Schematic illustration of the experimental timeline for assessing the in vivo biosafety of HVs or HVs@HA-BR-KDEL. (**B**) IVIS bioluminescence images of normal and mild colitis mice at 12 and 24 hours after oral administration of HVs or HVs@HA-BR-KDEL. (**C**) Representative H&E-stained images of the stomach, small intestine, cecum, and colon from mice following oral gavage of nanovesicles for 4 consecutive days. Scale bars, 100 μm. (**D**) Ex vivo bioluminescence images of major organs and the GI tract harvested from mice at 24 hours postadministration of HVs or HVs@HA-BR-KDEL. (**E**) Ex vivo bioluminescence images and (**F**) quantification of fluorescence intensity in the intestinal tracts of mice at 24 hours after oral gavage of HVs or HVs@HA-BR-KDEL (*n* = 4). (**G**) Representative immunofluorescence images showing the colocalization of CD11b (red) and DiD-labeled nanovesicles (green) in colon tissue sections, with nuclei stained with DAPI (blue). Scale bars, 100 μm. Data are presented as the means ± SD. *P < 0.05; **P < 0.01; ***P < 0.001.

The in vivo biosafety of HVs@HA-BR-KDEL was assessed in mice that were gavaged with HVs@HA-BR-KDEL (1.5 × 10^11^ particles/kg; 100 μl day^−1^) for 4 days (Fig. 5A). A 4-day repeated gavage protocol was selected for this short-term toxicity study in accordance with well-established methods for assessing acute oral toxicity (*52*, *53*). This duration allows for sufficient nanovesicles accumulation in tissues, enabling the detection of both measurable biological effects and potential early-onset inflammatory responses. Histological examination of GI tissues and major organs (heart, liver, spleen, lung, and kidney) via hematoxylin and eosin (H&E) staining revealed no apparent signs of tissue damage or inflammatory cell infiltration on day 5, following a 4-day oral administration with phosphate-buffered saline (PBS), HVs, or HVs@HA-BR-KDEL (Fig. 5C and fig. S14). Furthermore, blood biochemical analysis showed that the levels of routine blood indices in mice treated with HVs or HVs@HA-BR-KDEL remained comparable to those in the PBS control group (fig. S15). Collectively, these results demonstrate the excellent in vivo biosafety of the developed nanovesicles.

The stability and biodistribution of HVs@HA-BR-KDEL in vivo was investigated using both healthy mice and a mild colitis model. The mild colitis was induced via a 4-day administration of 5% (w/v) dextran sulfate sodium (DSS), in a protocol adapted from a previous study and validated in our pilot studies to yield a mild phenotype (*54*). This phenotype was characterized by a loss of 3 to 6% of initial body weight and a disease activity index (DAI) of 2 to 3 according to preliminary experiments. At the mild colitis stage, the tissue would be sufficiently inflamed to up-regulate CD44 for active targeting by our HA-modified nanovesicles, but not so severely damaged that nonspecific accumulation due to enhanced permeability and retention (EPR) effects would dominate. In this way, an accurate assessment of active ligand-receptor–mediated targeting rather than passive accumulation was achieved. To directly visualize and compare the biodistribution of nanovesicles, we performed near-infrared fluorescence (NIRF) imaging. Specifically, 1,1′-dioctadecyl-3,3,3′,3′-tetramethylindotricarbocyaine iodide (DiR) (excitation/emission = 748/780 nm)–labeled HVs or HVs@HA-BR-KDEL was orally administered to healthy and mild colitis mice for 12 and 24 hours. The 12-hour time point was chosen to ensure sufficient exposure and interaction of the nanovesicles with the inflamed colon site, thereby capturing their initial biodistribution. The 24-hour time point was chosen to distinguish active CD44 receptor-mediated retention from passive accumulation by allowing time for nonspecifically bound nanovesicles to be cleared and to assess targeting specificity. The fluorescence images of DiR-labeled nanovesicles were acquired by an in vivo imaging system (IVIS). Fluorescence signals in the abdomens of mice persisted for 12 hours after oral gavage but decreased markedly by 24 hours (Fig. 5B). Moreover, the signals were strongest in the HVs@HA-BR-KDEL (DSS) group at both time points, whereas HVs@HA-BR-KDEL (normal) and HV (DSS) groups showed markedly lower intensity. Following ex vivo imaging and quantitative analysis of intestinal tissues collected at 24 hours (Fig. 5, E and F), the mean fluorescence intensity in the HVs@HA-BR-KDEL (DSS) group remained significantly higher than that in the other two groups (*P* < 0.001), confirming the superior ability of HVs@HA-BR-KDEL to target and accumulate within inflamed intestinal tissues. In addition, colocalization analysis of the 1,1′-dioctadecyl-3,3,3′,3′-tetramethylindodicarbocyanine perchlorate (DiD)–labeled HVs@HA-BR-KDEL (depicted as green) with CD11b (depicted as red), a general marker for inflammatory cells, was performed via immunofluorescence staining to verify efficient uptake by inflammatory cells in the inflamed colon. As shown by fluorescence imaging (Fig. 5G), the absence of yellow fluorescence in the normal colon treated with HVs@HA-BR-KDEL indicated a lack of colocalization and thus negligible nonspecific uptake. Within the DSS-induced inflamed colon, a substantial infiltration of CD11b^+^ cells was observed. Administration of nontargeted HVs led to a modest signal increase but showed only weak colocalization with these cells. Notably, in the inflamed colon treated with the targeted HVs@HA-BR-KDEL, an intense green signal was detected that extensively overlapped with CD11b^+^ staining, producing a prominent yellow signal in the merged images. This clear colocalization confirms that HVs@HA-BR-KDEL is highly effective at promoting the uptake by the desired inflammatory cells in the diseased colon tissues. On the basis of this targeted efficacy, we further evaluated the biodistribution of orally administered HVs@HA-BR-KDEL in major organs (heart, liver, spleen, lungs, and kidneys) and the GI tract. The results demonstrated that the fluorescence signals were exclusively confined to the intestinal tract after 24-hour treatment, with no accumulation detected in other major organs, indicating minimal systemic absorption and a reduced risk of off-target side effects (Fig. 5D). Notably, the signal intensity in the HVs@HA-BR-KDEL (DSS) group was significantly stronger than that in the HV (DSS) group or the HVs@HA-BR-KDEL (normal) group. This observation suggests that the enhanced localized retention may involve the active targeting mechanism mediated by HA, which specifically binds to CD44 receptors up-regulated in the inflamed intestinal tissues.

To confirm successful siRNA delivery to the intestine, mild colitis mice were orally administered HVs@HA-BR-KDEL encapsulating FAM-siRNA. Mild colitis mice treated with HVs and healthy mice treated with HVs@HA-BR-KDEL were used as controls. At 24 hours, strong FAM-siRNA signals were observed specifically in the small intestine, cecum, and colon of the HVs@HA-BR-KDEL (DSS) group (fig. S16). In contrast, markedly weaker signals were detected in both HVs (DSS) and HVs@HA-BR-KDEL (normal) groups. These findings demonstrate that HVs@HA-BR-KDEL effectively traversed the GI tract and targeted the inflamed intestinal tissues.

### Therapeutic efficacy of HVs@HA-BR-KDEL against colitis in mice

**Fig. 6.**
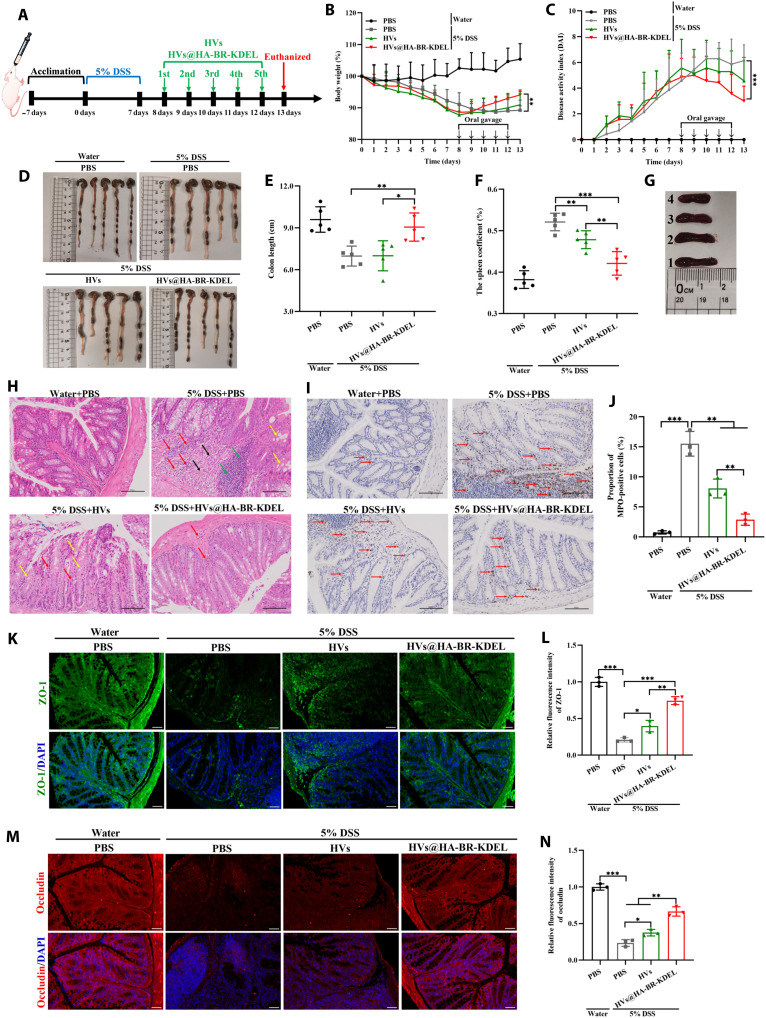
Therapeutic efficacy of orally administered HVs@HA-BR-KDEL in murine colitis. (**A**) Schematic illustration of the experimental design for treatment. (**B**) Daily body weight change of mice for 13 days (*n* = 5). (**C**) DAI of mice during the treatment (*n* = 5). (**D**) Representative macroscopic images and (**E**) corresponding quantified lengths of harvested colons (*n* = 5). (**F**) Spleen coefficient (*n* = 5) and (**G**) representative harvested spleen images of mice. 1 to 4: splenic shapes of the colitis model, HVs, HVs@HA-BR-KDEL, and healthy mice groups. (**H**) Representative H&E staining images of colon tissues. Scale bars, 100 μm. (**I**) Representative MPO staining images of colon tissues. Scale bars, 100 μm. (**J**) Quantification of MPO-positive cell proportion in colon tissues (*n* = 3). (**K** and **M**) Representative immunofluorescence staining images of the ZO-1 (K) and occludin (M) in the colon sections. The nuclei were stained with DAPI (blue). Scale bars, 50 μm. (**L** and **N**) Quantitative fluorescence intensity of ZO-1 (L) and occludin (N) expression in colon sections (*n* = 3). Data are presented as the means ± SD. *P < 0.05; **P < 0.01; ***P < 0.001.

The DSS-induced colitis mice model was established by dosing 5% DSS in drinking water for 7 days. Subsequently, the mice were orally gavaged daily for 5 consecutive days with HVs@HA-BR-KDEL [at a dose of 1.5 × 10^11^ particles/kg, containing siRNA (200 μg/kg)]. Mice with colitis that were orally administered PBS, sEPVs, siRNA-OMVs, or nontargeted HVs served as the control groups. The mice were lastly euthanized, and their colonic tissues were collected for analysis (Fig. 6A and fig. S17A). As shown in Fig. 6B, during the recovery phase, mice treated with HVs@HA-BR-KDEL regained body weight more rapidly than those in other groups. The HV group also exhibited a trend of weight recovery but at a slower rate compared to the HVs@HA-BR-KDEL group. In contrast, the colitis model group showed persistently impaired weight gain throughout the recovery period (*P* < 0.01 versus the HVs@HA-BR-KDEL group). Similarly, both the sEPVs and siRNA-OMV groups showed an impairment in weight recovery comparable to the model group, with no significant improvement observed (fig. S17C). Assessment of DAI revealed that treatment with HVs@HA-BR-KDEL resulted in a highly significant reduction (*P* < 0.001 versus model; Fig. 6C), whereas siRNA-OMVs induced a moderate but significant alleviation of symptoms (*P* < 0.05 versus model; fig. S17E). However, neither the sEPVs nor the nontargeted HVs achieved a statistically significant improvement. Consistent with its superior DAI performance, the HVs@HA-BR-KDEL group exhibited a colon length comparable to healthy control and significantly longer than the model and HV groups (Fig. 6, D and E), confirming the therapeutic efficacy. In contrast, the colon lengths in the siRNA-OMV, sEPV, and HV groups were similar to that in the model group although a statistical difference was observed in the siRNA-OMV group relative to the model control (*P* < 0.01) (fig. S17, B and D). In the DSS-induced colitis model, systemic inflammation often leads to splenomegaly. The spleen weight, normalized to body weight as the spleen coefficient, serves as a reliable indirect indicator of inflammation severity (*55*). The HVs@HA-BR-KDEL group exhibited a 19.2% decrease (*P* < 0.001) in this coefficient compared to the model group and a 11.9% decrease (*P* < 0.01) compared to the HV group. Along with this reduction, the splenic shape was restored to a state similar to that of healthy mice (Fig. 6, F and G). Although these integrated phenotypic and organ-level metrics confirm the superior efficacy of the HVs@HA-BR-KDEL, they may not fully capture the direct, mechanistic actions at the primary disease site. Therefore, to obtain a definitive and quantitative assessment of therapeutic potency, we performed direct histopathological and biochemical analyses of the colon tissues. These tissue-level evaluations were critical to determine the capacity of each vesicular formulation to resolve local inflammation, counteract oxidative stress, and promote cellular repair.

Representative H&E-stained colon sections revealed distinct histological features across experimental groups. Healthy mice exhibited intact colonic epithelium, abundant goblet cells, and tightly arranged crypts (Fig. 6H). Conversely, the colitis model group exhibited severe and extensive histological damage, characterized by profound disruption of the mucosal architecture. This was evidenced by ulceration featuring loss of intestinal glands replaced by proliferating granulation and fibrous connective tissue (black arrow), along with a marked reduction in goblet cells (yellow arrow) and irregular morphology of adjacent glands in these areas. Furthermore, the ulcerated and thickened mucosa showed pronounced inflammatory cell infiltration (red arrow), comprising numerous pus cells at the luminal surface (blue arrow) and a dense infiltration of lymphocytes within the expanded submucosa (green arrow) (Fig. 6H and fig. S17F). Critically, neither sEPV nor siRNA-OMV treatment improved histopathology as both groups exhibited core pathological features of the colitis model. These included ulceration with gland necrosis and replacement by granulation tissue (black arrow), pronounced inflammatory infiltration (red arrow), a marked reduction in goblet cells (yellow arrow), and extension into a thickened, inflamed submucosa (green arrow) (fig. S17F). In contrast, HV treatment showed a partial yet distinct therapeutic effect. Although damage was alleviated compared to the model group, features such as epithelial cell shedding (blue arrow), persistent albeit reduced inflammatory infiltration (red arrow), and reduced goblet cells (yellow arrow) indicated incomplete repair (Fig. 6H). HVs@HA-BR-KDEL treatment demonstrated a clear advancement, markedly protecting colon integrity by substantially reducing inflammation, preserving goblet cells, and promoting tightly arranged crypt structures (Fig. 6H). Myeloperoxidase (MPO) activity, a marker of neutrophil infiltration, was markedly increased in the model group (*P* < 0.001 versus healthy control) but was significantly attenuated by siRNA-OMV (34% reduction), HV (48%), and HVs@HA-BR-KDEL (81%) treatments, with the latter being the most effective (Fig. 6, I and J, and fig. S17, H and I). Although siRNA-OMVs reduced acute inflammation (MPO), they failed to improve histological architecture (H&E staining), underscoring the structural repair limitation of relying solely on EcN-derived OMVs. In the HV platform, the OMVs provide a physical barrier against GI degradation, whereas EGC palmitate both enhances the intrinsic stability of the siRNA complex and contributes inherent reparative bioactivities. The ultimate formulation, HVs@HA-BR-KDEL, synergistically combined targeted anti-inflammatory action with enhanced siRNA delivery, thereby achieving concurrent MPO reduction and profound histological restoration.

The alcian blue/periodic acid–Schiff (AB/PAS) staining results revealed that HVs@HA-BR-KDEL treatment preserved goblet cell numbers and restored the mucus layer to an extent comparable to the healthy group (fig. S18). Specifically, compared to the healthy group, goblet cell numbers per mm decreased by ~74 and 52% in the model and HV groups, respectively. In contrast, the HVs@HA-BR-KDEL group effectively preserved the goblet cell numbers. In addition, according to the immunofluorescence staining images (Fig. 6, K to N), DSS treatment notably reduced the expression of ZO-1 and occludin—two tight junction–related proteins that play crucial roles in maintaining intestinal epithelial function and mucosal barrier integrity (*56*). As expected, administration of HVs@HA-BR-KDEL to DSS-colitis mice restored the expression patterns of ZO-1 and occludin. Their expression levels were remarkably higher than those in the colitis model (ZO-1: 3.6-fold; occludin: 2.8-fold) and HVs (ZO-1: 1.9-fold; occludin: 1.8-fold) groups, demonstrating the great alleviation effect of HVs@HA-BR-KDEL on DSS-induced colitis.

The high concentration of ROS in the pathological microenvironment of IBD causes severe damage to the colon epithelium barrier (*57*). To assess the ROS scavenging effect of HVs@HA-BR-KDEL, we performed dihydroethidium (DHE) staining in DSS-colitis mice. As shown in fig. S19, DSS treatment markedly elevated ROS levels, which were 6.5-fold higher than those in the healthy group. Whereas sEPVs showed no significant effect (fig. S20, A and B), siRNA-OMVs significantly reduced ROS versus both the model and sEPV groups (*P* < 0.05). Furthermore, the quantitative reduction in ROS relative to the model group was ranked as follows: siRNA-OMVs (23%), HVs (41%), and HVs@HA-BR-KDEL (64%), with statistically significant differences observed among these three effective treatments (fig. S20C). Notably, HVs@HA-BR-KDEL treatment was the most effective, lowering ROS levels to only 2.3 times those of the healthy group (fig. S19B). To directly assess the efficacy of *TNF-*α siRNA delivery and the resulting gene silencing, we quantified its mRNA expression in colonic tissues. Whereas DSS-colitis markedly up-regulated *TNF-*α mRNA expression versus healthy controls (*P* < 0.001) (figs. S21 and S22A), OMV-based formulations significantly attenuated this increase in a treatment-dependent manner. Specifically, siRNA-OMVs, HVs, and HVs@HA-BR-KDEL achieved reductions of 8, 26, and 41%, respectively, with significant differences among them (fig. S22B). In contrast, the non-OMV formulation (sEPVs) showed no effect. The therapeutic failure of sEPVs establishes that OMVs are critical protective barriers for siRNA in the harsh GI environment. Conversely, the superior efficacy of HVs over siRNA-OMVs demonstrates the indispensable role of EGC palmitate, which enhances the intrinsic stability of the siRNA complex and contributes reparative bioactivities. Thus, the hybrid design synergistically integrates the protective function of OMVs with the stabilizing and bioactive properties of EGC palmitate, which collectively enable sufficient bioactive delivery to the site of inflammation. Building on this foundation, by further integrating active targeting, HVs@HA-BR-KDEL achieved the greatest suppression of *TNF-*α mRNA. This successful on-target gene silencing confirms the effective delivery of functional siRNA to the inflamed colon, which is consistent with the observed amelioration of colitis symptoms and pathology. Having established these local effects, we next evaluated whether the therapy also mitigated systemic inflammation by quantifying inflammatory cytokines in serum. As shown in fig. S23, the model group exhibited significant up-regulation of proinflammatory cytokines including TNF-α, IL-1β, and IL-6, as well as a down-regulation of anti-inflammatory IL-10. As expected, after treatment with HVs@HA-BR-KDEL, the levels of these proinflammatory cytokines were remarkably reduced (*P* < 0.001), whereas the level of IL-10 was obviously increased (*P* < 0.001). Notably, the modulatory effect of HVs@HA-BR-KDEL treatment on all tested inflammatory cytokines was significantly greater than that of HV treatment alone (*P* < 0.001), illustrating its superior anti-inflammatory efficacy. Furthermore, to investigate the role of HVs@HA-BR-KDEL in modulating ER stress in a DSS-colitis model, the expression of two key ER stress markers, CHOP and GRP78, was assessed in colonic tissues by immunohistochemistry (IHC) and ELISA. IHC analysis revealed a significant increase in the abundance of CHOP-positive (2.9-fold increase) and GRP78-positive cells (7.4-fold increase) following 5% DSS challenge (fig. S24). Treatment with HVs@HA-BR-KDEL markedly attenuated this up-regulation, resulting in reductions of ~50 and 29% for CHOP and GRP78, respectively, compared to the model group (*P* < 0.001), and 38 and 18% compared to the HV group (*P* < 0.001). Consistent with the IHC findings, ELISA quantification confirmed the potent inhibitory effect of HVs@HA-BR-KDEL on colitis-induced CHOP and GRP78 overexpression (fig. S25).

Dose-response studies are fundamental for defining the therapeutic window of a drug by elucidating its efficacy profile across a range of concentrations (*58*). To fully characterize the dose-response relationship of HVs@HA-BR-KDEL following its promising efficacy at a single dose (200 μg/kg siRNA), we conducted a systematic in vivo dose-ranging study. Specifically, mice were administered HVs@HA-BR-KDEL via oral gavage at siRNA doses ranging from 50 to 600 μg/kg daily for 5 days (fig. S26A). Body weight recovery during treatment exhibited a clear dose-dependent response (fig. S26B). At lower doses (50 and 100 μg/kg), the body weight remained stable without further decline. In contrast, doses of 200 μg/kg and above actively promoted weight gain, whereas the colitis model group showed persistently impaired weight gain throughout the recovery period. Quantification of this effect, calculated as [(weight at end of treatment − nadir weight)/(initial healthy weight − nadir weight)] × 100%, revealed that the body weight recovery percentage was significantly higher than the model group from the dose of 100 μg/kg onward (fig. S26F), with the doses of 400 and 600 μg/kg achieving notable recovery percentages of 25 and 30%, respectively. Treatment also significantly preserved colon length at all doses compared to the model group (fig. S26, C to E and G). However, unlike the graded weight response, colon protection reached a plateau at much lower doses, showing no significant differences among treatment groups (50 to 600 μg/kg). Dose-response fitting confirmed this, yielding a low median effective concentration (EC_50_) of 50.2 μg/kg, with the maximal effect within the range of 100–600 μg/kg (fig. S26H). In addition, clinical disease activity, assessed by the DAI, was significantly reduced at all doses from 50 μg/kg (fig. S26I). The dose-response relationship for DAI followed a classic sigmoidal curve, with a median inhibitory concentration (IC_50_) of 220.0 μg/kg (fig. S26J). The response plateaued at doses of ≥400 μg/kg, achieving the lowest DAI, which indicated superior clinical remission. Last, to directly confirm the gene silencing efficacy of our siRNA delivery system in the target tissue, we quantified *TNF-*α mRNA expression in colonic tissues. HVs@HA-BR-KDEL significantly suppressed colonic *TNF-*α mRNA expression from 50 μg/kg onward (fig. S27A), achieving maximal suppression of 71 to 74% at the two highest doses tested. This response yielded an IC_50_ of 209.8 μg/kg and was approaching a plateau at 400 μg/kg (fig. S27B). Therefore, our multilevel pharmacodynamic analysis revealed a stratified therapeutic response: Colon length preservation was the most sensitive endpoint (EC_50_ ~ 50.2 μg/kg), followed by *TNF-*α mRNA silencing (IC_50_ ~ 220 μg/kg) and DAI score improvement (IC_50_ ~ 209.8 μg/kg), which suggests that rapid structural repair may precede and facilitate systemic symptom resolution. Critically, the significant *TNF-*α knockdown and colon lengthening both occur at the same low dose, indicating they are closely linked. The apparent greater sensitivity of tissue repair likely reflects that the repair process can be efficiently triggered even by a partial reduction in the pro-inflammatory driver (*TNF-*α). In contrast, systemic recovery (body weight) displayed the most gradual, dose-proportional response, indicating that the complete restoration of metabolic homeostasis represents the ultimate challenge, requiring the highest level of therapeutic intervention.

Collectively, the excellent therapeutic efficacy of HVs@HA-BR-KDEL can be attributed to a synergy of its components: the GI stability of HVs, HA-mediated targeting of inflammatory cells in the inflamed tissues, KDEL-facilitated ER targeting, potent *TNF-*α silencing by siRNA, and the antioxidant effects of EGC palmitate and BR. This multifaceted mechanism underscores its great potential for treating intestinal-related diseases.

### Effects of HVs@HA-BR-KDEL on the gut microbiota

**Fig. 7.**
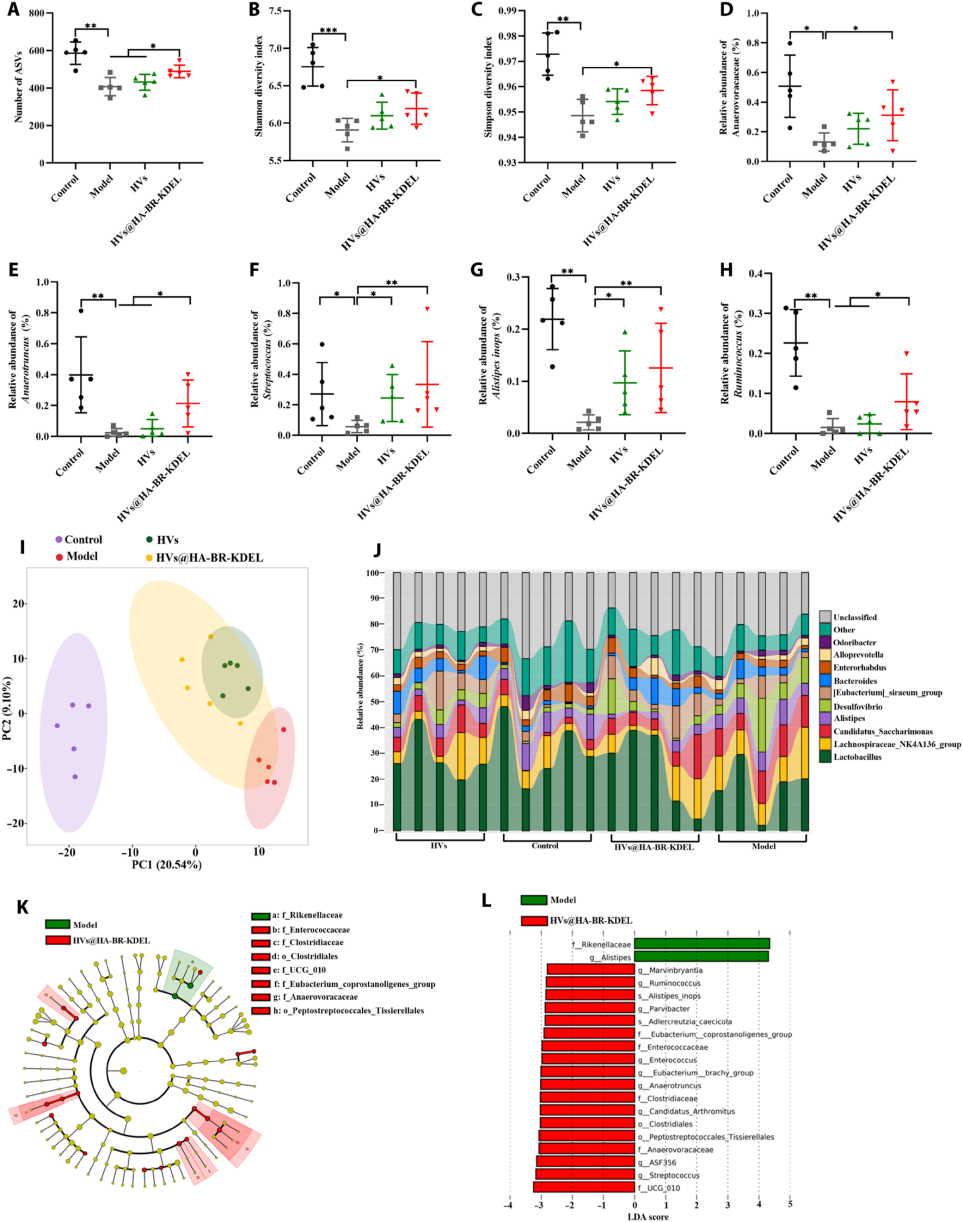
Alterations in the gut microbiota associated with HVs@HA-BR-KDEL treatment in colitis. (**A**) Number of observed ASVs in different experimental groups (*n* = 5). (**B** and **C**) Analysis of α-diversity of gut microbiota, determined by the (B) Shannon and (C) Simpson diversity index (*n* = 5). (**D**) Relative abundance of Anaerovoracaceae. (**E** to **H**) Relative abundance of (E) *Anaerotruncus*, (F) *Streptococcus*, (G) *A. inops*, and (H) *Ruminococcus*. (**I**) PCoA plot based on unweighted UniFrac distances showing the β-diversity of gut microbial communities. (**J**) Heatmap illustration of gut microbial distribution at the genus level. (**K**) LEfSe cladogram illustrating the differentially abundant bacterial lineages across groups. (**L**) Histogram of LDA scores for biomarkers with an LDA score greater than 2. The bar length corresponds to the effect size of each differentially abundant taxon.

Gut microbiota, the largest microbial ecosystem of human body, plays essential roles in maintenance of intestinal mucosal homeostasis, and IBD is strongly associated with the dysbiosis of gut microbiota (*59*). To determine whether HVs@HA-BR-KDEL treatment was associated with alterations in gut microbiota community structure, we profiled the fecal microbiota (composition and abundance) in a mouse model of colitis. BALB/c mice were treated as shown in Fig. 6A, and the fecal samples collected on day 13 were analyzed by 16*S* ribosomal RNA (rRNA) gene sequencing. Healthy mice given PBS, DSS-colitis mice without intervention, and DSS-colitis mice treated with HVs were used as controls. HVs@HA-BR-KDEL significantly increased the amplicon sequence variants (ASVs) richness and α-diversity (Shannon and Simpson indices) of the gut microbiota in colitis mice compared to the DSS-colitis model and HV groups (Fig. 7, A to C). To assess β-diversity (differences in microbial community composition between groups), we performed principal coordinate analysis (PCoA) based on unweighted UniFrac distance. The model group was distinctly separated from the healthy group, indicating a substantial shift in gut microbiota composition associated with colitis (Fig. 7I). In contrast, the HVs@HA-BR-KDEL group clustered closer to the healthy group, suggesting an association between the treatment and a mitigation of the colitis-induced dysbiosis.

Analysis of the community composition at finer taxonomic resolutions (family and genus) identified specific changes associated with treatment. Notably, the colitis model group exhibited a significant depletion of Anaerovoracaceae, a beneficial family known for its role in promoting the production of short-chain fatty acids (SCFAs), which are associated with gut health (*60*). Although treatment with the HVs alone showed a modest, nonsignificant trend of recovery, only HVs@HA-BR-KDEL treatment was associated with a statistically significant increase in the abundance of Anaerovoracaceae (*P* < 0.05), restoring it to levels comparable to those in healthy controls (Fig. 7D). These findings support the superior efficacy of HVs@HA-BR-KDEL and elucidate a potential mechanism by which it contributes to improved gut health, namely, through the restoration of beneficial SCFA-producing microbiota.

Beyond shifts at the family level, genus-level analysis revealed that HVs@HA-BR-KDEL treatment was correlated with selective enrichment of several beneficial bacterial genera with known protective functions (Fig. 7J and fig. S28). This included a significant increase in *Anaerotruncus* (a butyrate producer that aids in intestinal mucosa repair) (Fig. 7E) (*61*), *Streptococcus* (certain species are known to produce bacteriocins and lactate, contributing to pathogen inhibition and mucosal homeostasis) (Fig. 7F) (*62*), and *Ruminococcus* (degrades dietary fiber to produce acetate and succinate, which are used by other bacteria to enhance microbial community stability) (Fig. 7H) (*63*) Furthermore, the treatment was associated with the recovery of *Alistipes inops*, a species previously reported to have protective effects against colitis (*64*), which was reduced in the model group (Fig. 7G). Collectively, the restoration of these key taxa suggests a concerted shift toward a microbiota structure capable of promoting gut health and integrity. This restructuring of the microbial community was further supported by linear discriminant analysis effect size (LEfSe) analysis, which showed a clear separation between the model and treatment groups (Fig. 7, K and L, and fig. S29). These results revealed that the colitis model group was significantly enriched with pro-inflammatory gut microbiota, such as Enterobacteriaceae (elevated abundance is a marker of dysbiosis) (*65*) and *Escherichia-Shigella* (a virulent pathogen known to promote IBD) (*66*). Notably, the abundance of Enterobacteriaceae and *Escherichia-Shigella* was significantly decreased following HV treatment, and the HV group no longer exhibited strong disease-associated microbial characteristics but instead shifted toward commensal and neutral microbiota, such as *Muribaculum* and *Monoglobus* (fig. S29) (*67*). Moreover, HVs@HA-BR-KDEL treatment not only suppressed the expansion of pathogenic bacteria but also increased the abundance of beneficial bacteria such as Anaerovoracaceae and *Ruminococcus* (Fig. 7, K and L), a finding that was consistent with our prior results. Accordingly, treatment with HVs@HA-BR-KDEL ameliorated colitis symptoms and was accompanied by a mitigation of gut microbiota disorders, characterized by an increase in beneficial taxa.

## DISCUSSION

Naturally abundant polyphenols, which exhibit health-beneficial properties and a strong affinity for nucleic acids via hydrogen bonding and hydrophobic interactions, are promising building blocks for nucleic acid delivery vectors. Although various strategies, such as complexation, self-polymerization, and layer-by-layer assembly, have been exploited to create polyphenol-based carriers for RNA delivery, the direct encapsulation and formation of nano entities by polyphenols without assistance of additional materials remains unexplored. In this study, we developed a simple self-assembly strategy to efficiently encapsulate siRNA into nanovesicles using a specifically acylated, amphiphilic EGC derivative. Specific acylation of EGC precisely preserved the critical phenolic hydroxyl groups necessary for RNA affinity and antioxidant activity, demonstrating that specific modification via selective protection and deprotection approaches is valuable to finely tune the physicochemical properties of polyphenols without loss of beneficial functions (*11*, *12*, *68*). The EGC palmitate nanovesicle-based encapsulation represent a simple, safe, and efficient gene delivery platform that can potentially replace cytotoxic cationic liposomes.

Oral delivery of RNA is challenging but safe and convenient for IBD treatment to increase dosing concentration at the target site and minimize off-target administration. EcN is a well-established probiotic that is generally used for IBD treatment. The OMVs derived from EcN are great robust biocompatible carriers for oral delivery due to GI stability and ready availability, which also alleviate colitis and restore the intestinal homeostasis (*17*, *18*, *26*). As indicated in this study, the RNA encapsulated in the EGC palmitate nanovesicles could be efficiently transferred to OMVs through membrane fusion with simple sonication operation. Therefore, the vesicle-to-vesicle relayed transfer strategy substantially promoted the RNA delivery efficiency. Furthermore, OMVs can be readily anchored with different ligands for various functions. As demonstrated in this study, the anchoring HA-BR-KDEL targeting ligand successfully guided the OMVs for sequential cellular and intracellular targeting to ER of inflammatory cells in the intestinal tract. The sequential targeting strategy enhanced enrichment in the inflamed colon tissues and endocytosis of OMVs by inflammatory cells to the intracellular target site, which accordingly improved the overall treatment effect. Future work will explore the versatility of this platform for delivering other nucleic acid therapeutics, such as microRNA (miRNA) or mRNA, to expand its application potential for IBD and other GI diseases.

Our study demonstrates that HVs@HA-BR-KDEL effectively alleviates colitis and contributes to a more balanced gut microbiota, providing a multilayered understanding of its therapeutic mechanism. First, a dose-response study established the central role of *TNF-*α siRNA, providing clear evidence for its direct, sequence-specific action. The results show a dose-dependent knockdown of colonic *TNF-*α mRNA, with corresponding, graded improvements in disease activity, colon length, and body weight recovery. Mechanistically, this confirms that the therapeutic efficacy is directly tied to *TNF-*α siRNA delivery and silencing. Consequently, the microbiota restoration observed at the effective dose can be most reasonably explained as a downstream result of this siRNA-driven resolution of host inflammation. Second, functional deconvolution of the HV’s components through controlled in vivo comparisons revealed that both OMVs and EGC palmitate are indispensable. OMVs serve as a critical protective barrier for siRNA during GI transit, whereas EGC palmitate enhances the complex’s stability and contributes complementary bioactivities, together enabling the platform’s superior performance. Last, the consistent co-occurrence of inflammation resolution and microbial restoration at the effective dose provides a coherent mechanistic narrative. We acknowledge that definitive causal dissection remains a valuable future direction. To this end, we propose an experimental roadmap using germ-free mice colonized with a defined microbial community. Comparing the outcome of our nanovesicles with that of a conventional anti-inflammatory drug in this setting will yield clear, interpretable results. A unique microbiota shift induced by our vesicles would indicate a direct remodeling effect, whereas identical shifts dependent solely on inflammation suppression would confirm a secondary consequence. Any intermediate result will help precisely quantify the contribution of direct versus indirect mechanisms.

In summary, we established an efficient strategy for siRNA encapsulation and stabilization using HVs derived from amphiphilic EGC palmitate and EcN-derived OMVs, followed by functionalization with HA-BR-KDEL. On the basis of our findings, the alleviation of colitis by HVs@HA-BR-KDEL involves multiple specific active components and underlying molecular mechanisms. The system relies on *TNF-*α siRNA as the core therapeutic agent to silence the pro-inflammatory cytokine gene. It achieves targeted accumulation at inflamed sites through HA-mediated binding to CD44 receptors, which are up-regulated on inflammatory colon tissues. Following this, the system is internalized via clathrin-mediated endocytosis, whereas the incorporated KDEL peptide further facilitates ER targeting, promoting efficient siRNA release and cytosolic delivery. In addition, the EGC palmitate and BR components contribute to ROS scavenging. Furthermore, these processes collectively support the amelioration of microbial dysbiosis. Specifically, EcN-derived OMVs may modulate host immunity including interacting with Toll-like receptors (TLRs) to reduce inflammation, indirectly supporting beneficial bacteria (*69*). Meanwhile, EGC palmitate and BR components alleviate oxidative stress, creating a favorable environment for commensal microbes. Thus, gut microbiota restoration appears to be a beneficial consequence of reduced inflammation and improved barrier function. Together, these components work synergistically to down-regulate inflammation, reduce oxidative stress, restore barrier integrity, and contribute to a more balanced gut microbiota, resulting in comprehensive colitis therapy.

## MATERIALS AND METHODS

### Materials

EGC, anhydrous acetonitrile, chloroform, anhydrous dimethyl sulfoxide (DMSO), propionic anhydride, hydroxylamine solution (50 wt % in H_2_O), 1-ethyl-3-(3-dimethylaminopropyl)carbodiimide hydrochloride (EDC·HCl), chlorpromazine, and amiloride were from Shanghai Aladdin Biochemical Technology Co. Ltd. Triethylamine, pyridine, palmitoyl chloride, HA (100 ~ 200 kDa), BR, and FITC were from Shanghai Macklin Biochemical Co. Ltd. Anhydrous dichloromethane, petroleum ether, ethyl acetate, and 95% ethanol were from Sinopharm Chemical Reagent Co. Ltd. *N*-Hydroxysuccinimide (NHS) was from Shanghai Meryer Co. Ltd. Nystatin was from Shanghai Yuanye Bio-Technology Co. Ltd. The KDEL peptide was synthesized by Nanjing GenScript Biotechnology Co. Ltd. *TNF-*α siRNA (sense: 5′-CACAACCAACUAGUGGUGCUU-3′; antisense: 5′-AAGCACCACUAGUUGGUUGUG-3′) and FAM-labeled *TNF-*α siRNA were synthesized by Shanghai Sangon Biotech Co. Ltd. DiO, DiI, DiD, and DiR were purchased from Nantong Feiyu Biological Technology Co. Ltd. Biological materials included the following: EcN (from our laboratory’s stock), RAW264.7 cells (Hangzhou Leyi Biotechnology Co. Ltd.), Luria-Bertani (LB) medium (Beijing Coolaber Science & Technology Co. Ltd.), Dulbecco’s modified Eagle’s medium (DMEM) (Dalian MeilunBio Co. Ltd.), and fetal bovine serum (FBS) (Beijing Solarbio Science & Technology Co. Ltd.). Assay kits and antibodies were sourced from Shanghai Beyotime Biotechnology Co. Ltd. [CCK-8, ROS assay kit, calcein/propidium iodide (PI) live/dead viability assay kit, NO detection kit, ER-Tracker Red probe, and Hoechst 33342 dye], Wuhan Pinuofei Co. Ltd. (antibodies against CD11b, MPO, occludin, ZO-1, CHOP, and GRP78), Shanghai Jianglai Biological Co. Ltd. (high-sensitivity ELISA kits for TNF-α, IL-6, IL-10, IL-1β, CHOP, and GRP78), and BD Biosciences (San Jose, CA, USA) (FITC-conjugated anti-CD44 antibody). All reagents were used as received without any further purification, unless otherwise stated.

### Synthesis of EGC palmitate

#### Protection of phenolic hydroxyl groups

Propionic anhydride (131.5 μl, 1.0 mmol) and triethylamine (138.6 μl, 1.0 mmol) were added dropwise to a solution of EGC (61.2 mg, 0.2 mmol) in anhydrous acetonitrile (2 ml). The resultant mixture was stirred at RT for 4 hours and monitored by TLC with petroleum ether/ethyl acetate (1:2, v/v). Subsequently, the reaction mixture was extracted with ethyl acetate, washed twice with deionized (DI) water, followed by dehydration using anhydrous magnesium sulfate. After filtration, rotary evaporation was carried out to remove ethyl acetate and then protected EGC products were obtained.

#### Esterification of protected EGC

Pyridine (24.2 μl, 0.3 mmol) was added dropwise to a solution of protected EGC (117.2 mg, 0.2 mmol) in anhydrous dichloromethane (2 ml), and then palmitoyl chloride (125.8 μl, 0.4 mmol) was added dropwise. The reaction mixture was stirred for 30 min at RT and monitored by TLC with petroleum ether/ethyl acetate (1:2, v/v), after which the protected EGC palmitate was obtained for further use. To remove excess palmitoyl chloride, 95% ethanol (2 ml) was added to the mixture. The reaction was then continued for another 2 hours.

#### Deprotection of protected EGC palmitate

Hydroxylamine (122.5 μl, 2.0 mmol) was added dropwise to the reaction mixture obtained above, stirred for 2 hours at RT, and monitored by TLC with petroleum ether/ethyl acetate (1:2, v/v). Then, the reaction solution was extracted with ethyl acetate and washed twice with aqueous solution of hydrochloric acid (1 M) and DI water in sequence, followed by dehydration using anhydrous magnesium sulfate. After filtration, the organic phase was collected and evaporated. Last, the obtained solid product was suspended in petroleum ether, filtered, and dried, yielding a product of EGC palmitate (96.5 mg; yield, 88.6%).

#### Structural characterization of EGC palmitate

The ^1^H NMR spectra of the synthesized EGC palmitate were collected on a 400-MHz NMR spectrometer (Bruker Corporation, Switzerland) using DMSO-*d*_6_ as solvent. The HPLC-MS analysis of EGC palmitate was performed on an Agilent 1200 system (Agilent, Santa Clara, CA, USA) with an Agilent ZORBAX Eclipse XDB-C18 (150 mm by 2.1 mm, 3.5 μm) column. The column temperature was maintained at 40°C, and the injection volume was 20 μl. The mobile phase for HPLC-MS comprised 0.1% aqueous solution of formic acid (A) and acetonitrile (B). The gradient elution profile started with 95% A. After 5 min, B was gradually increased to 90% within 20 min and then maintained for 5 min. The mobile phase was delivered at a flow rate of 1.0 ml/min, and signals were monitored at 272 nm with Diode Array Detector (DAD) detection. Mass spectra were obtained using a Thermo Finnigan LCQ Deca XP Max system (Thermo Fisher Scientific, USA) in ESI mode with a scan range of *m/z* 200 to 2000.

### Preparation of sEPVs

Before the preparation of sEPVs, EGC palmitate was dissolved in 70% (v/v) ethanol as a stock solution. For vesicle formation, *TNF-*α siRNA (1.5 μg in 500 μl of nuclease-free water) was mixed with an equal volume of 70% ethanol, followed by brief sonication (200 W, 2 min) in a water bath to ensure homogeneity. Subsequently, the EGC palmitate (90 μg) was added dropwise to the siRNA mixture under magnetic stirring, after which the mixture was sonicated (200 W) for 2 min and then continuously stirred at RT for 1 hour to form sEPVs. To remove unencapsulated siRNA and free EGC palmitate, centrifugation was performed at 10,000*g* for 5 min at 4°C using a 50-kDa molecular weight cutoff (MWCO) centrifugal ultrafiltration device (Millipore, USA). The retentate containing the sEPVs was washed with nuclease-free water, followed by three repeated cycles of ultrafiltration to remove excess ethanol and free molecules. The purified sEPVs were lastly suspended in nuclease-free water and stored at 4°C for further use.

### Isolation of OMVs derived from EcN

OMVs were isolated from EcN as previously described (*70*). Briefly, a single colony was inoculated into LB medium and grown overnight at 37°C. A 200-μl aliquot of this culture was transferred into 1600 ml of fresh LB broth and incubated overnight at 37°C with shaking at 220 rpm. Bacterial cells were then removed from the culture by centrifugation at 12,000*g* for 3 min at 4°C. The obtained supernatant was filtered through a 0.45-μm sterile filter (Millipore, USA) and concentrated by ultrafiltration membranes with an MWCO of 100 kDa (Millipore, USA). OMVs were collected by ultracentrifugation at 150,000*g* for 2 hours at 4°C, washed once with PBS, resuspended in PBS, and lastly stored at −80°C until use.

### Synthesis of HA-BR-KDEL

Before synthesizing HA-BR-KDEL, the HA-BR conjugate was prepared as previously described (*22*, *23*). BR was modified to form an aminoethylene-BR conjugate (AE-BR). In brief, BR (0.1 mmol) was dissolved in 2 ml of anhydrous DMSO and NHS (0.1 mmol) was added dropwise to the solution. After stirring for a few seconds, EDC (0.1 mmol) was added dropwise to the reaction mixture and stirred for 30 min at 37°C under nitrogen gas. Subsequently, ethylenediamine (0.3 mmol) was added dropwise to the above solution and then stirred under nitrogen protection for 1 hour and monitored by TLC. Then, the mixture was extracted with chloroform. The organic layer was then collected, washed twice with DI water twice, dehydrated, filtered, and evaporated. The solid product was suspended in methanol and centrifuged at 3000*g* for 10 min to remove free BR, after which the supernatant was evaporated to yield AE-BR. To synthesize the HA-BR conjugate, HA (12 mg) and NHS (0.1 mmol) were mixed in 4 ml of DMSO. After EDC (0.1 mmol) was dropwise added, the reaction mixture was stirred for 30 min at 37°C. Then, AE-BR (0.1 mmol) was added, and the solution was stirred overnight at RT under nitrogen gas. Dialysis was conducted against DI water for 24 hours, which was changed every 4 hours. The resulting solution was lastly lyophilized, yielding HA-BR.

The preparation of HA-BR-KDEL adopted the reported protocol with slight modification (*71*). HA-BR (12 mg) and NHS (0.1 mmol) were mixed in 4 ml of PBS. After EDC (0.1 mmol) was dropwise added, the reaction mixture was stirred for 30 min at 37°C. Subsequently, KDEL (0.1 mmol) was dropwise added to the reaction mixture and reacted overnight maintaining pH 7 to 8 at 37°C, followed by dialysis against DI water for 24 hours. The HA-BR-KDEL product was obtained via lyophilization and then was stored at −20°C for further use. The successful synthesis of HA-BR-KDEL was confirmed by ^1^H NMR analysis.

### Preparation of HVs and HVs@HA-BR-KDEL

On the basis of the structural similarity between sEPVs and OMVs, the HVs were produced by membrane fusion via sonication (*72*, *73*). Briefly, sEPVs and OMVs, each at a concentration of 2.0 × 10^9^ particles/ml, were mixed in equal volumes (200 μl each) to a final volume of 400 μl. The mixture was sonicated using an all-in-one ultrasonic cell disruptor (Xiaomei ultrasonic instrument, China) with the following parameters: 30% amplitude and cycles of 5-s on and 5-s off, for a total of 24 cycles. During this step, the sample was in an ice bath to avoid destruction of membrane components caused by heat. Subsequently, the sonicated mixture was gently shaken for 1 hour at 37°C to allow for membrane recovery, yielding the final HVs. The same sonication parameters were applied to the preparation of OMVs encapsulating siRNA (siRNA-OMVs). HVs@HA-BR-KDEL were prepared as described in the references with slight modification (*74*, *75*). Briefly, HA-BR-KDEL (60 μg) was added dropwise to 500 μl of HV suspension (8.0 × 10^8^ particles/ml) under magnetic stirring. After brief sonication (30% amplitude and cycles of 5-s on and 5-s off, for a total of 12 cycles) to transiently disrupt the membrane structure, the mixture was stirred for 30 min at RT for HVs@HA-BR-KDEL formation. To remove free HA-BR-KDEL, the mixture was centrifuged at 10,000*g* for 5 min at 4°C using a 50-kDa MWCO ultrafiltration device. The retentate was collected and resuspended in PBS to yield the final HVs@HA-BR-KDEL.

### Characterization of nanovesicles

#### Measurement of TNF-α siRNA loading efficiency

The siRNA loading efficiency of sEPVs and HVs was assessed by agarose gel electrophoresis, as described previously with slight modifications (*9*, *30*). Briefly, prepared sEPVs were purified by centrifugal ultrafiltration (50-kDa MWCO) to remove unencapsulated siRNA, excess EGC palmitate, and ethanol. The resulting retentate was then washed with nuclease-free water and subjected to three cycles of ultrafiltration to remove residual ethanol and free molecules. The final retentate was then analyzed by 2% agarose gel electrophoresis (100 V, 20 min). The siRNA loading efficiency was calculated as the ratio of the encapsulated siRNA band intensity (in the purified retentate) to the known band intensity of the initial naked siRNA input, with both samples at the same dilution. Quantification of band intensities was performed using ImageJ software (National Institutes of Health, USA). To optimize the formulation, different weight ratios of siRNA to EGC palmitate (1:20, 1:40, 1:60, and 1:80) were tested. Similarly, after preparation of HVs and siRNA-OMVs, the same ultrafiltration method was used to separate unloaded siRNA from the centrifugal permeate. For these samples, loading efficiency was inversely estimated by collecting the permeate and analyzing it via 2% agarose gel electrophoresis.

#### CLSM, TEM, FRET, NTA, and DLS assays

The morphology and structural features of sEPVs and HVs were characterized by CLSM and TEM. Briefly, sEPVs were prepared using FAM-siRNA and EGC palmitate. To visualize the vesicle membrane, the prepared sEPVs were then labeled with the lipophilic fluorescent dye DiI by incubation. Following incubation, free dye was removed by centrifugation at 10,000*g* for 5 min at 4°C using a 50-kDa MWCO ultrafiltration device. The colocalization of the green fluorescence from FAM-siRNA (indicating the encapsulated cargo) and the red fluorescence from DiI (indicating the vesicle membrane) was observed by CLSM (Leica TCS-SP8, Germany). Similarly, to validate the membrane fusion, HVs were prepared using sEPVs (encapsulating FAM-siRNA) and OMVs (labeled with DiI) and then visualized by CLSM. A simple physical mixture of the sEPVs and OMVs without sonication served as the control. Membrane fusion was further confirmed by a FRET-based assay. In this process, sEPVs and OMVs were separately labeled with the FRET pair DiO (donor) and DiI (acceptor) at a concentration of 4 μg/ml. The labeled nanovesicles were fused via sonication. The fluorescence emission spectrum of the fused product was measured from 500 to 620 nm (excitation at 460 nm) using a fluorescence spectrophotometer (TECAN, Switzerland). In addition, CLSM was used to verify the successful anchoring of FITC-labeled HA-BR-KDEL into the hydrophobic layer of DiI-labeled HVs. The morphologies of nanovesicles were analyzed via TEM (HitachiH-7650, Japan) after negative staining. In brief, a drop of nanovesicles suspension was adsorbed for 2 min on Formvar/carbon-coated grids previously activated by ultraviolet (UV) light. Then, grids were washed with DI water, stained with 2% uranyl acetate for 2 min, air dried, and observed by TEM. To analyze the particle number and size of nanovesicles, NTA measurement was carried out on a NanoSight NS500 (Malvern Instruments, UK). The zeta potential was measured by DLS Zetasizer Nano ZS90 (Malvern, UK).

#### Stability assays in vitro

The stability of sEPVs and HVs was evaluated by RNase and SGF degradation assays (*8*, *76*). For the RNase degradation assay, preprepared sEPVs and HVs were used. Subsequently, the naked siRNA, sEPVs, and HVs were separately incubated with RNase A (2 ng/μl) for 5 min, after which the enzymatic degradation was blocked by an RNase inhibitor. The reaction solutions were lastly sampled to detect the siRNA bands by 2% agarose gel electrophoresis, followed by calculation of the degradation degree of siRNA using the ImageJ software. To evaluate stability in SGF, 100 μl of naked siRNA, sEPVs, and HVs were separately mixed with 10 μl of SGF (pH 1.2) and incubated at 37°C for 2 hours. After incubation, the acidic reaction was neutralized to terminate degradation. The samples were then analyzed by 2% agarose gel electrophoresis to assess the protective effect of the nanovesicles, and the degradation rate was calculated.

### Cell viability assay

For the CCK-8 assay, RAW264.7 cells were seeded in 96-well plates at a density of 5.0 × 10^3^ cells per well and cultured in DMEM supplemented with 10% FBS at 37°C in a 5% CO_2_ incubator for 24 hours. The medium was then replaced with 100 μl of fresh medium containing HVs@HA-BR-KDEL at varied initial feeding concentrations in terms of siRNA (5, 10, 15, 20, 25, and 30 μg/ml). After 24 hours of incubation, the medium was removed, and the cells were washed twice with PBS. Subsequently, 100 μl of fresh medium containing 10% CCK-8 solution was added to each well, followed by incubation for 2 hours. The optical density at 450 nm (OD_450_) values were recorded using a microplate reader (TECAN, Switzerland). For the live/dead staining assay, RAW264.7 cells were seeded in 24-well plates at 1.0 × 10^5^ cells per well and cultured overnight. The cells were then treated with HVs@HA-BR-KDEL (4.0 × 10^8^ particles/ml) for 24 hours. After treatment, the cells were washed with PBS and stained with calcein acetoxymethyl ester (calcein AM) and PI for 30 min at 37°C. The characteristic fluorescence of cells was observed by CLSM after washing the cells twice with PBS.

### Cellular uptake analysis

To investigate the active CD44 receptor-targeting ability of HA-BR-KDEL, cellular uptake of various nanovesicles was evaluated in RAW264.7 cells modeled with LPS-induced inflammation. Briefly, cells were seeded in 24-well plates (2.0 × 10^5^ cells per well) for 24 hours, followed by stimulation with LPS (200 ng/ml) for 12 hours to establish the inflammatory model. The cells were then treated for 6 to 24 hours with naked FAM-siRNA or an equivalent FAM-siRNA dose (3.0 μg/ml) encapsulated in sEPVs, OMVs, HVs, or HVs@HA-BR-KDEL (all at 4.0 × 10^9^ particles/ml). Untreated cells served as the control. After treatment, the cells were stained with Hoechst 33342 and washed with PBS, followed by immediate observation by CLSM. For quantification, the cells were gently scraped, resuspended in PBS, and the cellular uptake was analyzed using a flow cytometer (Beckman Coulter, USA).

To confirm CD44 overexpression in LPS-induced inflammatory RAW264.7 cells, a flow cytometry–based antibody binding assay was performed. Briefly, cells were seeded in 24-well plates (2.0 × 10^5^ cells per well) and cultured for 24 hours. The medium was then replaced with high-glucose DMEM containing LPS (200 ng/ml). After 12 hours of stimulation, the cells were washed twice with ice-cold PBS and incubated with a FITC-conjugated anti-CD44 antibody (5 μg/ml) for 2 hours at 4°C in the dark. Normal cells with antibody treatment served as the control. Following the incubation, unbound antibody was removed by washing, and the cells were harvested for flow cytometric analysis. To further investigate the contribution of the HA-CD44 targeting pathway to cellular uptake, cells were pretreated with an anti-CD44 antibody (5 μg/ml) for 2 hours at 4°C to block the receptors before the addition of DiI-labeled HVs@HA-BR-KDEL. Cells incubated with HVs@HA-BR-KDEL alone and untreated cells served as positive and negative controls, respectively. After 24-hour treatment, cells were washed and collected in PBS, and DiI fluorescence was measured by flow cytometry.

To investigate the ER-targeting ability of HA-BR-KDEL, RAW264.7 cells were treated with HVs, HVs@HA-BR, or HVs@HA-BR-KDEL for 12 to 24 hours as described above. Subsequently, the cells were stained with ER-Tracker Red for 30 min at 37°C. After removing the free ER-Tracker Red, the cells were stained with Hoechst 33342 and washed for observation by CLSM.

### Endocytosis pathway study

RAW264.7 cells were seeded in 24-well plates (2.0 × 10^5^ cells per well) and cultured overnight to achieve above 80% coverage. The cells were then washed with PBS and pretreated with chlorpromazine (5 μg/ml), nystatin (5 μg/ml), or amiloride (100 μg/ml) at 37°C for 1 hour. Subsequently, the cells were treated with HVs@HA-BR-KDEL encapsulating FAM-siRNA (4.0 × 10^9^ particles/ml) at 37°C for 12 hours. After incubation, the cells were harvested, and the cellular fluorescence intensity was analyzed qualitatively by CLSM and quantitatively by flow cytometry.

### Anti-inflammatory activity in vitro

RAW264.7 cells were seeded in 24-well plates (2.0 × 10^5^ cells per well) and incubated for 24 hours. The culture medium was then replaced with high-glucose DMEM containing LPS (200 ng/ml) alone or in combination with siRNA (3.0 μg/ml), sEPVs (4.0 × 10^9^ particles/ml), siRNA-OMVs (4.0 × 10^9^ particles/ml), HVs (4.0 × 10^9^ particles/ml), or HVs@HA-BR-KDEL (4.0 × 10^9^ particles/ml). Cells treated with medium only served as the negative control. After 24 hours of incubation, the supernatant was collected. The concentration of NO was measured using a commercial NO detection kit, whereas the levels of cytokines (TNF-α, IL-6, and IL-10) were determined by respective ELISA kits according to the manufacturer’s protocols. Furthermore, two marker proteins of ER stress (CHOP and GRP78) were tested by ELISA kits after collecting the adherent cells in each well.

### Oxidative stress assay

RAW264.7 cells were seeded in 24-well plates (2.0 × 10^5^ cells per well) and incubated for 24 hours. The culture medium was then replaced with high-glucose DMEM containing LPS (200 ng/ml) alone or in combination with siRNA (3.0 μg/ml), sEPVs (4.0 × 10^9^ particles/ml), siRNA-OMVs (4.0 × 10^9^ particles/ml), HVs (4.0 × 10^9^ particles/ml), or HVs@HA-BR-KDEL (4.0 × 10^9^ particles/ml) for an additional 24 hours. Cells treated with medium only served as the negative control. To detect intracellular ROS levels, the cells were washed with PBS and incubated with ROS-sensitive probe DCFH-DA for 30 min at 37°C. After washing twice with PBS to remove excess probe, the cells were processed for two parallel analyses. For qualitative observation, the cells were immediately imaged by CLSM. For quantitative analysis, the cells were gently scraped, collected in PBS, and analyzed by flow cytometry.

### In vivo biosafety and biodistribution analysis

Female BALB/c mice aged 8 weeks were obtained from the Hangzhou Medical College Laboratory Animal Center (Hangzhou, China). All animal experiments were reviewed and approved by the Institutional Animal Care and Use Committee, Zhejiang Center of Laboratory Animals (ZJCLA) (ethical number ZJCLA-IACUC-20010925).

The in vivo biosafety of HVs@HA-BR-KDEL was evaluated in mice. Briefly, after a 7-day acclimatization period, nine mice were randomly divided into three groups (*n* = 3). Two treatment groups received a daily oral gavage of either HVs or HVs@HA-BR-KDEL (1.5 × 10^11^ particles/kg in 100 μl of PBS; *TNF-*α siRNA payload: 200 μg/kg) for 4 consecutive days. The third group, administered with PBS only, served as the control. On the fifth day, all mice were euthanized. Blood samples were collected, and the serum was separated by centrifugation at 2000 rpm for 10 min for biochemical analysis. Major organs, including the heart, liver, spleen, lungs, kidneys, stomach, small intestine, cecum, and colon, were harvested. These organs were fixed in 4% paraformaldehyde, embedded in paraffin, and sectioned for H&E staining to assess histopathological changes.

The biodistribution and inflammatory tissue targeting of the formulation were evaluated in vivo using DiR-labeled HVs@HA-BR-KDEL. Briefly, 12 mice were randomly divided into three groups (*n* = 4) after a 7-day acclimatization period. Mild colitis was induced in two groups by administering 5% DSS (Bide Pharmatech, Shanghai, China) in drinking water for 4 days. The third group of healthy mice served as a control. Subsequently, each mouse received an oral gavage (100 μl of PBS) of the following preparations: Colitis model group 1 was given DiR-labeled HVs@HA-BR-KDEL (1.5 × 10^11^ particles/kg; *TNF-*α siRNA payload: 200 μg/kg), colitis model group 2 received an equivalent dose of DiR-labeled HVs, and the healthy control group received DiR-labeled HVs@HA-BR-KDEL. Whole-body fluorescence imaging was performed using an IVIS spectrum system (PerkinElmer, Lumina LT) at 12 and 24 hours postadministration. Furthermore, mice were euthanized at 24 hours, and the GI tract and other major organs were harvested for ex vivo imaging to quantify the fluorescence signals.

To analyze the uptake of FAM-siRNA in the GI tract, mild colitis mice received an oral administration of HVs@HA-BR-KDEL as described above. At 24 hours postadministration, intestinal tissues were harvested, processed for paraffin embedding, and sectioned for observation by CLSM.

### Therapeutic efficacy against DSS-induced colitis

To evaluate the therapeutic efficacy of HVs@HA-BR-KDEL, mice were acclimatized for 7 days and then randomly divided into six groups before formal experiments. Acute colitis was induced in five of the groups by administering 5% (w/v) DSS in their drinking water ad libitum for 7 days, as previously described (*28*).

The sixth group, which served as the negative control, received normal drinking water throughout the experiment. After the 7-day DSS treatment, the modeling compound was replaced with normal drinking water. Subsequently, the five DSS-induced colitis groups received daily oral gavages for 5 consecutive days as follows: PBS (vehicle control), sEPVs, siRNA-OMVs, HVs, or HVs@HA-BR-KDEL (all at 1.5 × 10^11^ particles/kg with *TNF-*α siRNA payload of 200 μg/kg). The negative control group also received PBS. On the 13th day, the mice were euthanized. The body weight and DAI of each mouse were recorded daily over the 13-day experimental period. Blood, the whole colon tissues, fecal (colon site), and spleen samples were collected for further serological and histopathological analysis. The spleen coefficient is defined as the ratio of spleen weight (g) to mouse body weight (g). It was calculated using the following formula

$$\text{Spleen coefficient}=\frac{\text{Spleen weight}}{\text{Body weight}}\times 100\%$$

### Histopathology analysis

For H&E and AB/PAS staining, the colon tissue samples were fixed in 4% paraformaldehyde for 24 hours. Then, the distal colons were embedded in paraffin and sectioned for H&E and AB/PAS staining. The stained sections were examined under an optical microscope (Nikon, Japan).

### Immunohistochemical analysis

For immunohistochemical staining, distal colon tissues were paraffin embedded and sectioned at a thickness of 4 μm. After dewaxing and rehydration, antigen retrieval was performed by microwave heating in 0.01 M sodium citrate buffer. Endogenous peroxidases were then blocked by incubation with 3% hydrogen peroxide in methanol. Subsequently, the sections were blocked with 10% goat serum for 1 hour at RT to prevent nonspecific binding. The sections were then incubated overnight at 4°C with primary antibodies against MPO, CHOP, and GRP78. After washing, the sections were incubated with appropriate horseradish peroxidase (HRP)–conjugated secondary antibodies for 2 hours at RT. Signal visualization was achieved by staining with 3,3′-diaminobenzidine (DAB) for 10 min. Last, the sections were counterstained with hematoxylin, mounted with resin, and examined under a bright-field microscope (Nikon, Japan). The number of MPO-positive, CHOP-positive, and GRP78-positive cells (exhibiting brown DAB staining) was quantified using Image-Pro Plus 6.0 software (Media Cybernetics, USA).

### Immunofluorescence staining analysis

Immunofluorescence staining was performed on paraffin-embedded colon sections. After dewaxing and rehydration, the sections were washed with PBS, permeabilized, and blocked with PBS containing 0.3% Triton X-100 and 10% normal goat serum for 1 hour at RT. The sections were then incubated overnight at 4°C with primary antibodies against ZO-1 and occludin diluted in the blocking buffer. After washing three times with PBS, the sections were incubated with Cy3-conjugated secondary antibodies for 1 hour at RT. Following a final wash, the nuclei were counterstained with 4′,6-diamidino-2-phenylindole (DAPI). The slides were imaged by CLSM.

To analyze the colocalization of HVs@HA-BR-KDEL with inflammatory cells, frozen sections of colon tissue were fixed, permeabilized, and blocked. The sections were then incubated overnight at 4°C with an anti-CD11b antibody, followed by a Cy3-conjugated secondary antibody. Nuclei were counterstained with DAPI. The distribution of DiD-labeled nanovesicles and CD11b^+^ cells was visualized by CLSM.

### ROS staining

Intracellular ROS production in colon tissues was detected using DHE staining. Briefly, colon sections were incubated with DHE (Beyotime, China) for 30 min at 37°C in the dark. Following DHE staining, the nuclei were counterstained with DAPI for 20 min. Fluorescence images were acquired by CLSM, and fluorescence intensity was calculated by ImageJ.

### RT-qPCR assays

For the reverse transcription quantitative polymerase chain reaction (RT-qPCR) assays, RNA was extracted from cryopreserved colon tissues using TRIzol reagent (Beyotime, China) followed by isopropanol precipitation. RNA concentration and purity were measured by a NanoVue Plus spectrophotometer (Cytiva, USA). Purified RNA was then inversely transcribed into cDNA using the SweScript All-in-One RT SuperMix for qPCR (One-Step gDNA Remover) (Servicebio Co. Ltd., China) according to the manufacturer’s protocol. To eliminate genomic DNA contamination, no-RT controls (lacking reverse transcriptase) were run for all samples. Obtained cDNA was used as a template for RT-qPCR with SYBR Green to determine the expression of the *TNF-*α gene. Gene expression was normalized to the endogenous reference gene *GAPDH*. RT-qPCR was performed using the 2× Universal Blue SYBR Green qPCR Master Mix (Servicebio Co. Ltd., China) on a fluorescence quantitative PCR instrument (Beijing Dongsheng Innovation Biotechnology, China). Each 20-μl reaction contained 10 μl of Master Mix, 0.4 μl of each gene-specific forward and reverse primer (10 μM), and 2 μl of the cDNA template. Nuclease-free water was added to a final volume of 20 μl. The relative gene expression was calculated using the 2^−ΔΔCt^ method. The primer sequences used were as follows: *TNF-*α (forward, 5′-GATGGGTTGTACCTTGTCTACT-3′; reverse, 5′-CTTTCTCCTGGTATGAGATAGC-3′).

### Enzyme-linked immunosorbent assays

The levels of cytokines (TNF-α, IL-6, IL-10, and IL-1β) in the serum were quantified. Briefly, blood samples were centrifuged at 2000 rpm for 15 min at 4°C to obtain serum, which was then analyzed using commercial ELISA kits according to the manufacturer’s instructions. In addition, the levels of ER stress markers (CHOP and GRP78) in colon tissues were measured using corresponding ELISA kits.

### Gut microbiota analysis by 16*S* rRNA gene sequencing

After different treatments, feces collected from the colon samples on the 13th day were sent to Servicebio Co. Ltd. (Wuhan, China) for 16*S* rRNA gene sequencing. Following genomic DNA extraction, DNA concentration was quantified using a Qubit fluorometer (Thermo Fisher Scientific, USA), and the integrity of the extracted genomic DNA was assessed by 1.5% agarose gel electrophoresis. Then, the diluted DNA samples were amplified with barcoded primers (341F/805R) targeting the 16*S* rRNA V3-V4 region. The resulting PCR products were pooled, and sequencing adapters were ligated during library construction, followed by an additional PCR amplification step. The libraries were then purified with magnetic beads to remove primer dimers and other small fragments. All libraries were quantified using Qubit, and their index information was recorded. On the basis of these data, libraries were pooled in equimolar ratios and sequenced on an Illumina NovaSeq 6000 platform with a PE250 strategy.

The obtained raw demultiplexed FASTQ files were processed with DADA2 (v1.18.0) to generate ASVs. Briefly, raw forward and reverse reads were quality filtered (removing reads with ambiguous bases or expected error > 2) and quality trimmed (truncating at first base with *Q*-score < 2). Sample-specific error models were learned to denoise reads and infer ASVs. Denoised reads were merged [min. overlap: 12 base pairs (bp); zero mismatches], and chimeras were removed de novo. Subsequently, all diversity analyses were performed on a rarefied ASVs table. The α-diversity was assessed using the Shannon and Simpson indices, and the β-diversity was calculated on the basis of the unweighted UniFrac distance and visualized via PCoA. Differentially abundant taxa between groups were identified using the LEfSe tool on the Huttenhower Lab Galaxy server. The analysis used the Kruskal-Wallis test (*P* < 0.05) to detect significant abundance differences, followed by linear discriminant analysis (LDA) to estimate the effect size. Features with an LDA score greater than 2.0 (indicating a 10-fold effect size) were considered significant biomarkers.

### Statistical analysis

All experimental results were expressed as means ± SD (SD). Student’s *t* test and one-way analysis of variance (ANOVA) were performed in statistical significance analysis by GraphPad Prism 8.0 software. Statistical significance was expressed as *P < 0.05, **P < 0.01, and ***P < 0.001.
