# Supplementary material for: Targeted intracellular oral RNA delivery through tea polyphenol nanovesicle to outer membrane vesicle transfer for colitis treatment
Source: Sci Adv. 2026 Mar 27;12(13):eadx8336. doi: 10.1126/sciadv.adx8336 (PMC13025053; doi:10.1126/sciadv.adx8336)
Supplement: Supplementary file 1 — Figs. S1 to S29 [file sciadv.adx8336_sm.pdf]

Science Advances
AAAS

Supplementary Materials for

Targeted intracellular oral RNA delivery through tea polyphenol nanovesicle to outer membrane vesicle transfer for colitis treatment

Taisong Fang and Songbai Liu

Corresponding author: Songbai Liu, songbailiu@zju.edu.cn

Sci. Adv. **12**, eadx8336 (2026)
DOI: 10.1126/sciadv.adx8336

This PDF file includes:

Figs. S1 to S29

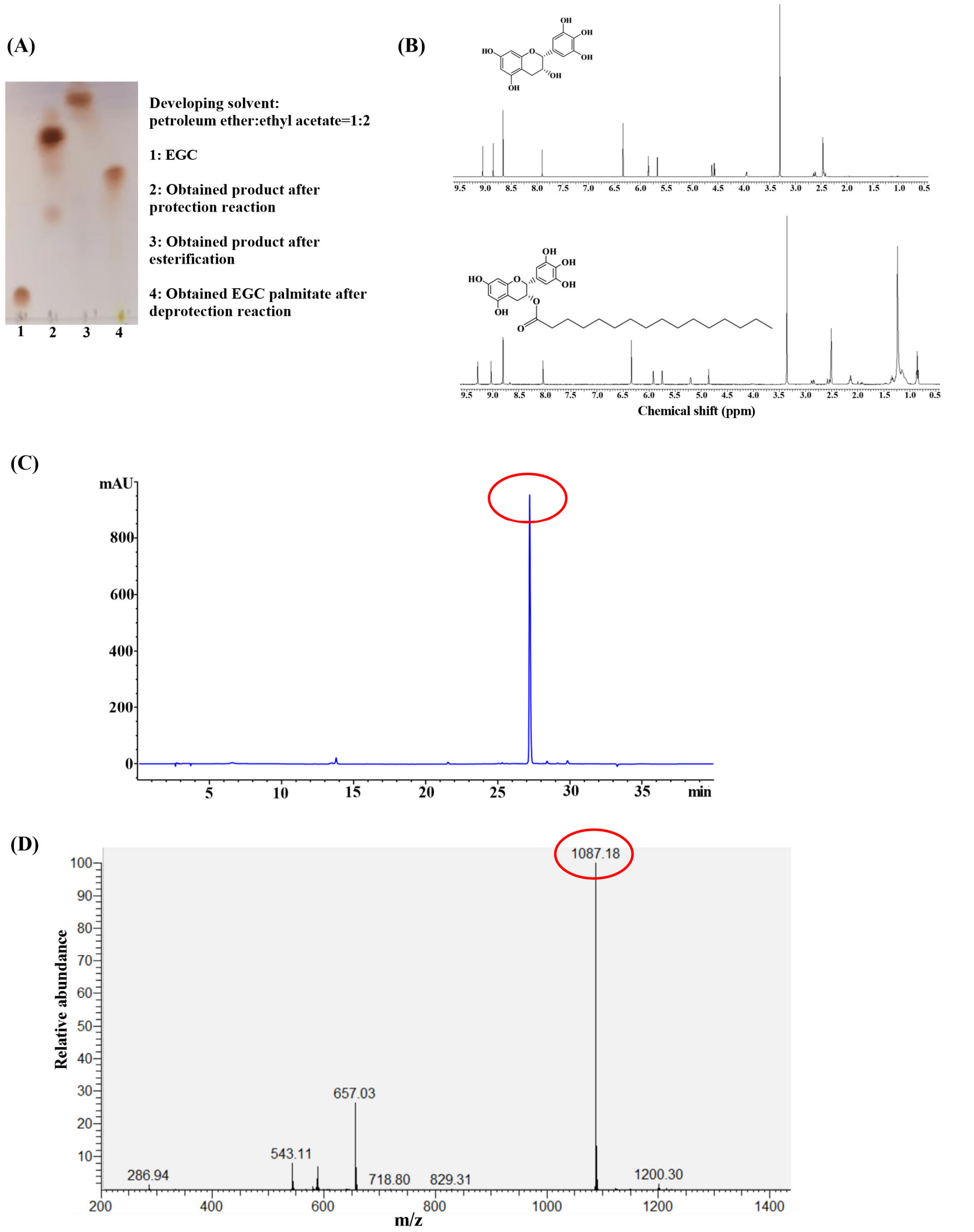

Fig. S1. Structural characterization of EGC palmitate. (A) TLC, **(B)** ^{1}H-NMR spectrum, **(C)** HPLC analysis, and **(D)** ESI-MS spectrum of EGC palmitate.

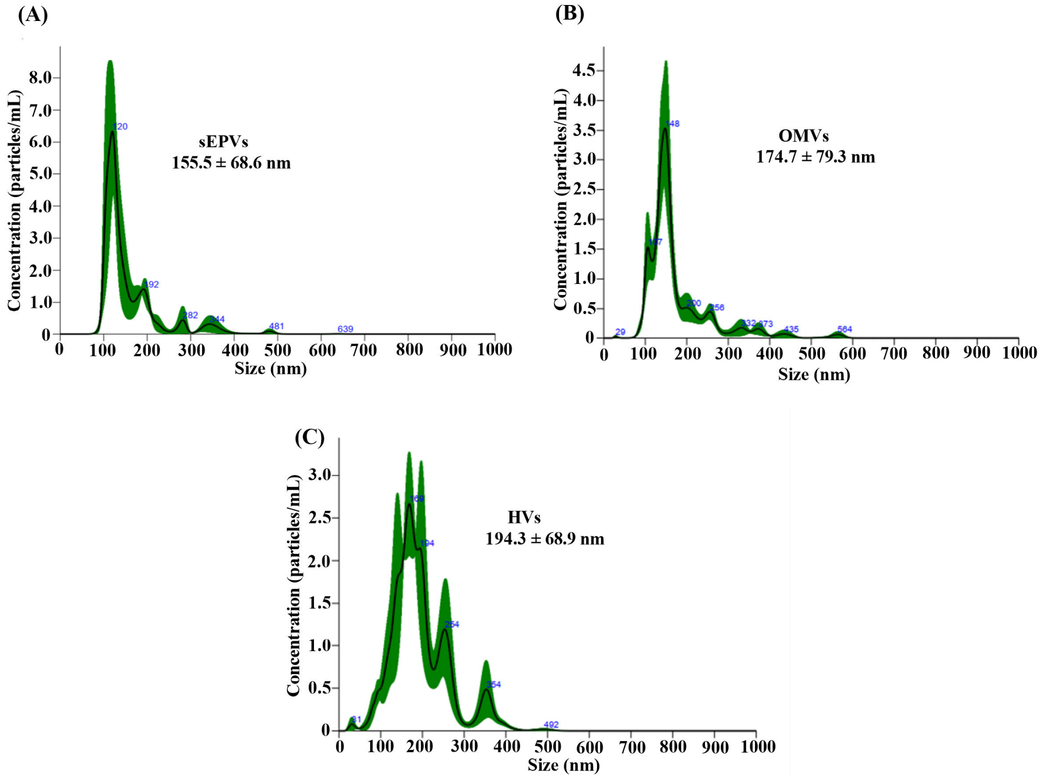

Fig. S2. Particle size distribution of nanovesicles measured by NTA. (A) sEPVs, **(B)** OMVs, and **(C)** HVs.

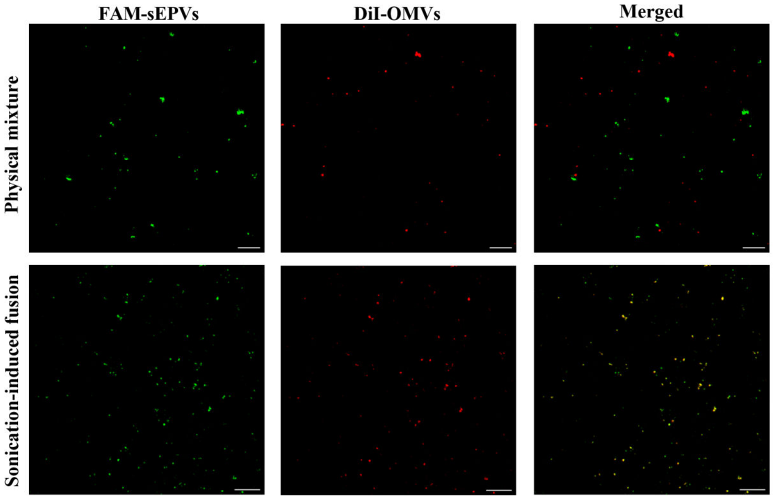

Fig. S3. Successful formation of HVs. CLSM images comparing the physical mixture of sEPVs and OMVs with the fused HVs. FAM-labeled siRNA (green) and DiI-labeled OMVs (red). Scale bar: 5 μm.

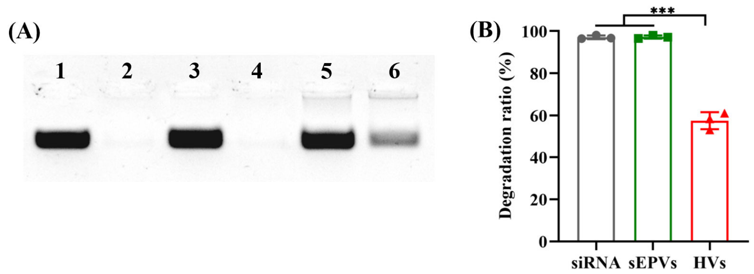

Fig. S4. Protection of siRNA against SGF degradation by nanovesicles. (A) Gel electrophoresis analysis of siRNA after exposure to SGF (37°C, 2 h). Lanes 1-2: naked siRNA (before/after SGF); Lanes 3-4: sEPVs (before/after SGF); Lanes 5-6: HVs (before/after SGF). **(B)** Quantitative analysis of degradation ratios (n=3). Data are presented as the means ± SD. *$p < 0.05$, **$p < 0.01$, ***$p < 0.001$.

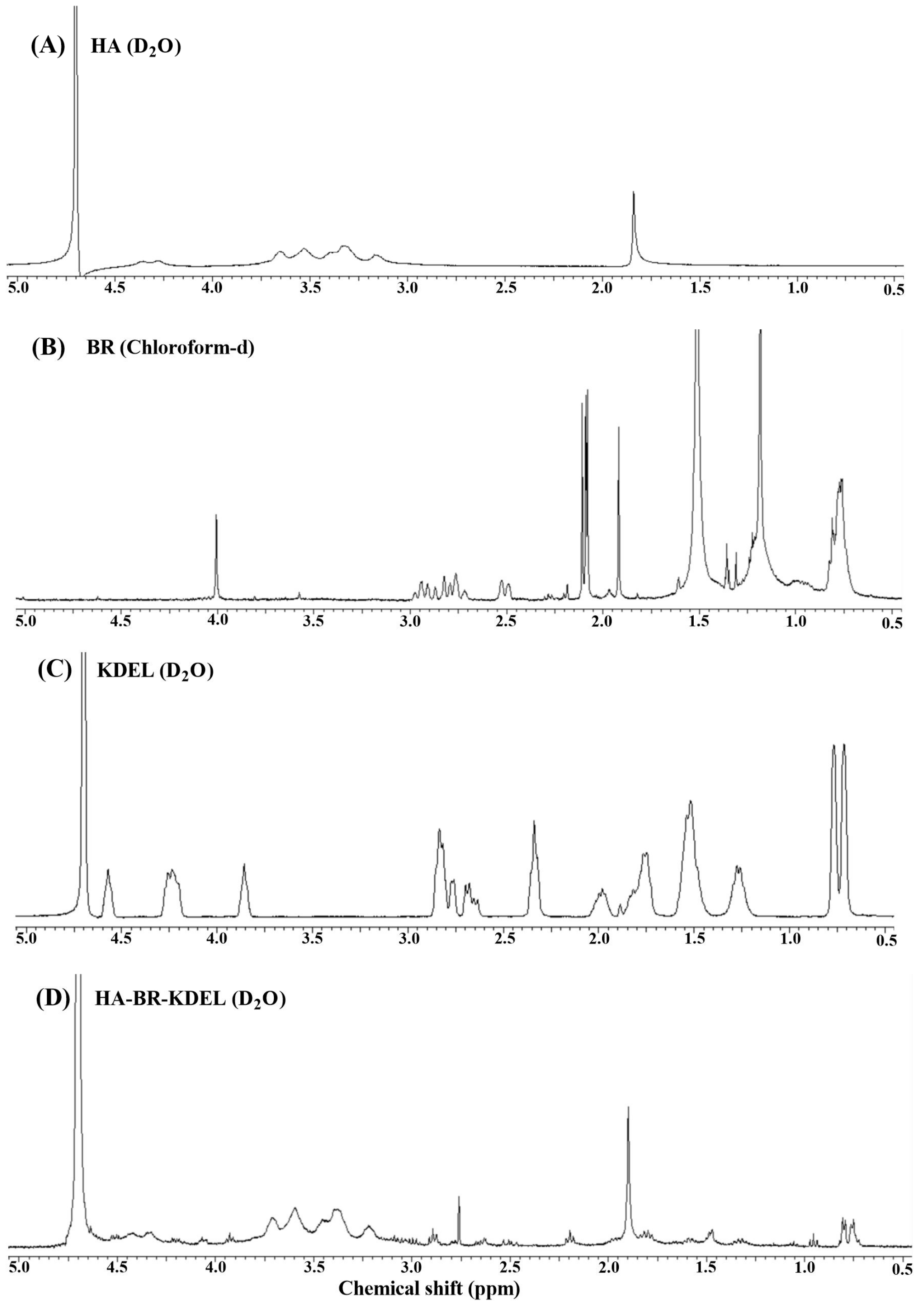

Fig. S5. ^{1}H-NMR spectra of the conjugate. (A) HA (D_2O), **(B)** BR (Chloroform-d), **(C)** KDEL (D_2O), and **(D)** the final HA-BR-KDEL conjugate (D_2O).

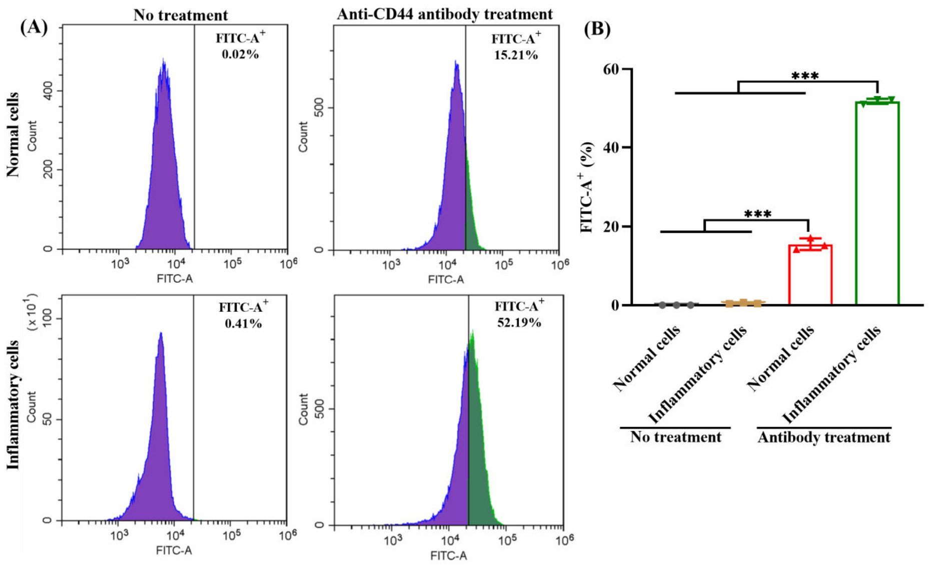

Fig. S6. Upregulation of CD44 expression in inflammatory RAW264.7 cells. (A) Flow cytometric analysis of CD44 expression in normal and inflammatory RAW264.7 cells. **(B)** Quantitative analysis of CD44 fluorescence intensity (n=3). Data are presented as the means ± SD. **p* < 0.05, ***p* < 0.01, ****p* < 0.001.

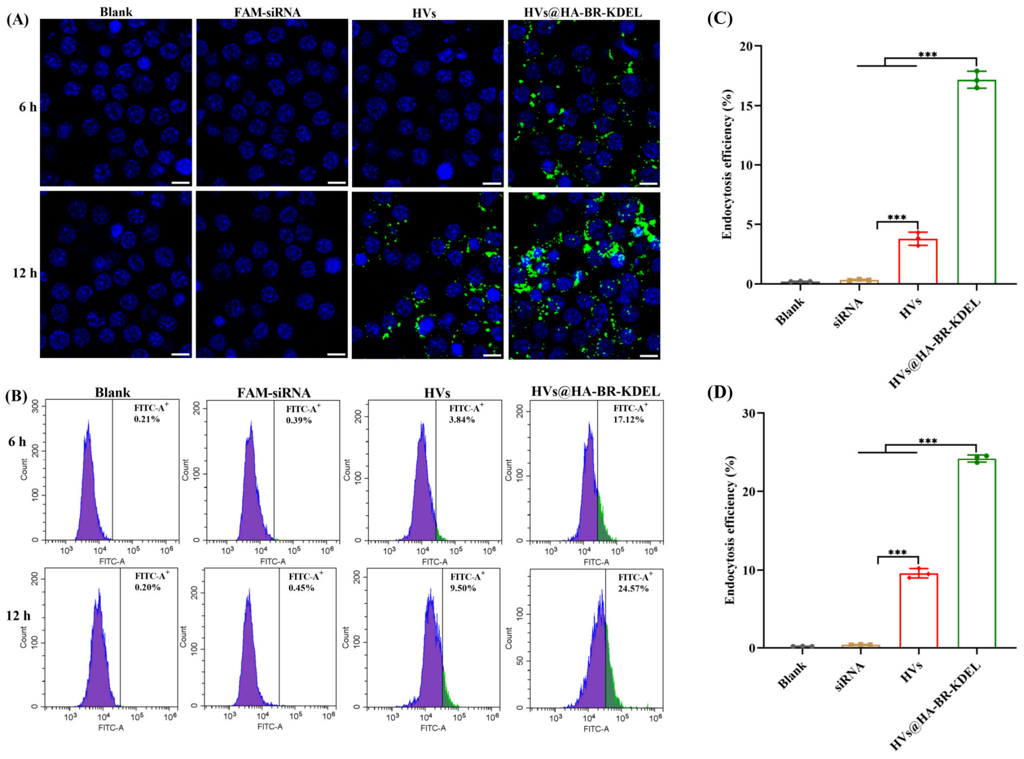

Fig. S7. Cellular uptake *in vitro*. (A) CLSM images and **(B)** Flow cytometric analysis of inflammatory RAW264.7 cells treated with naked or nanovesicles-encapsulated FAM-siRNA for 6 h and 12 h. Green: FAM-siRNA; Blue: Hoechst 33342 (nuclei). Scale bar: 10 μm. **(C)** and **(D)** Quantitative analysis of cellular uptake efficiency for 6

h (C) and 12 h (D) (n=3). Data are presented as the means ± SD. *$p < 0.05$, **$p < 0.01$, ***$p < 0.001$.

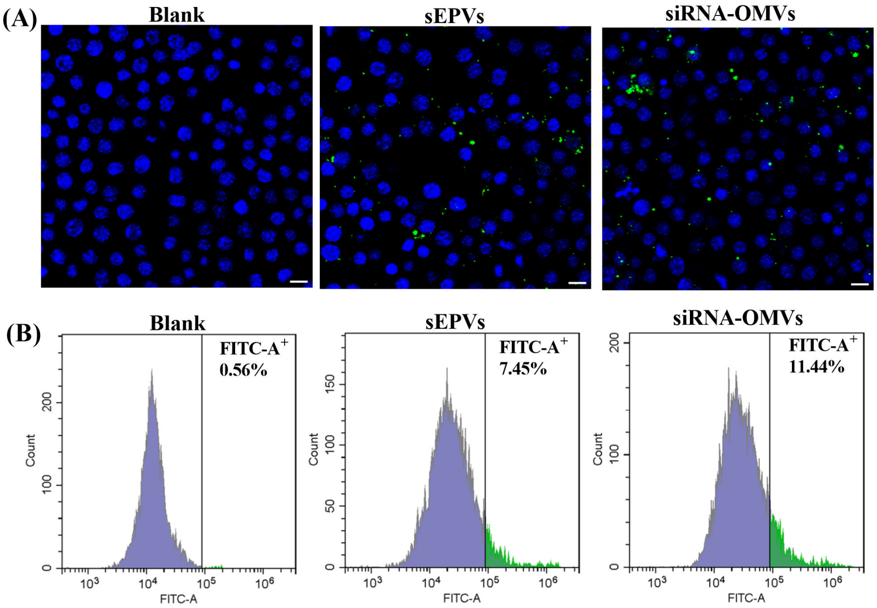

Fig. S8. Comparative analysis of cellular uptake mediated by sEPVs and OMVs *in vitro*. (A) CLSM images and **(B)** Flow cytometric analysis of inflammatory RAW264.7 cells after incubation with sEPVs or siRNA-OMVs for 24 h. Scale bar: 10 μm.

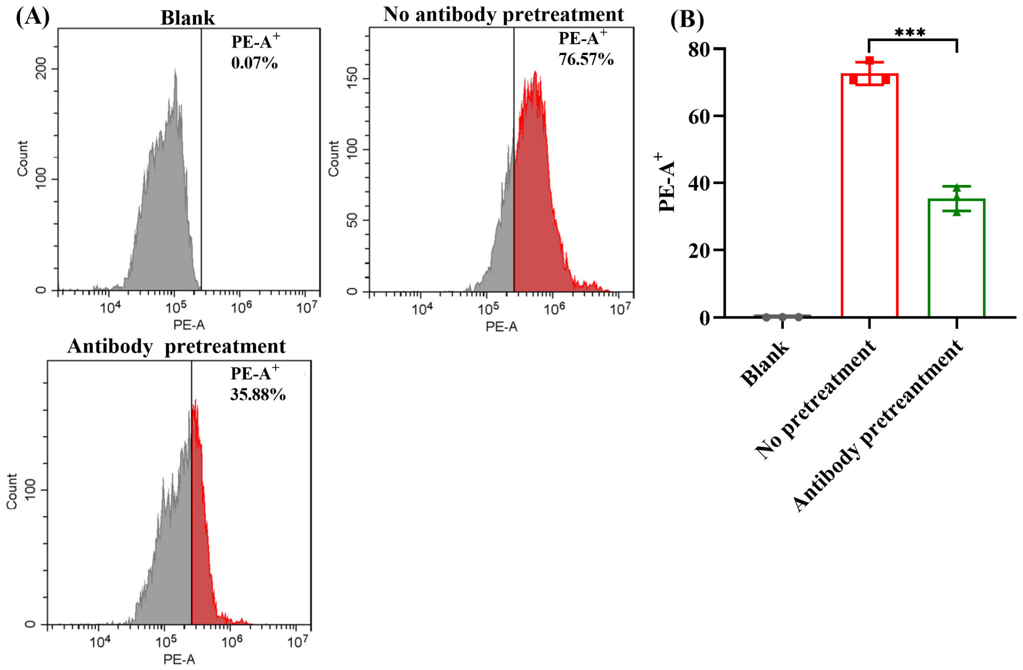

Fig. S9. The cellular uptake of HVs@HA-BR-KDEL is CD44-dependent. (A) Flow cytometric analysis of inflammatory RAW264.7 cells incubated with DiI-labeled HVs@HA-BR-KDEL following pretreatment with or without an anti-CD44 antibody (2 h, 4°C). **(B)** Quantification of DiI fluorescence intensity (n=3). Data are presented

as the means ± SD. *$p < 0.05$, **$p < 0.01$, ***$p < 0.001$.

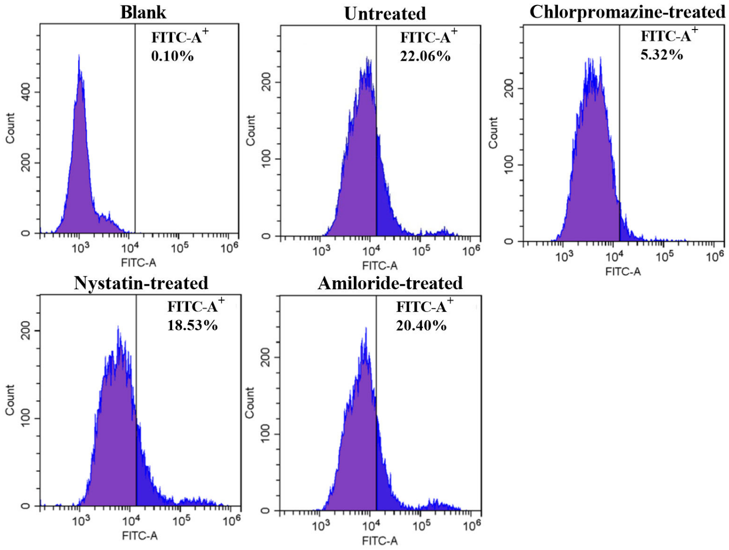

Fig. S10. Effect of endocytic inhibitors on HVs@HA-BR-KDEL uptake. Flow cytometric analysis of cellular uptake of HVs@HA-BR-KDEL after 12 h of incubation, with pretreatment of various endocytic inhibitors for 1 h at 37°C.

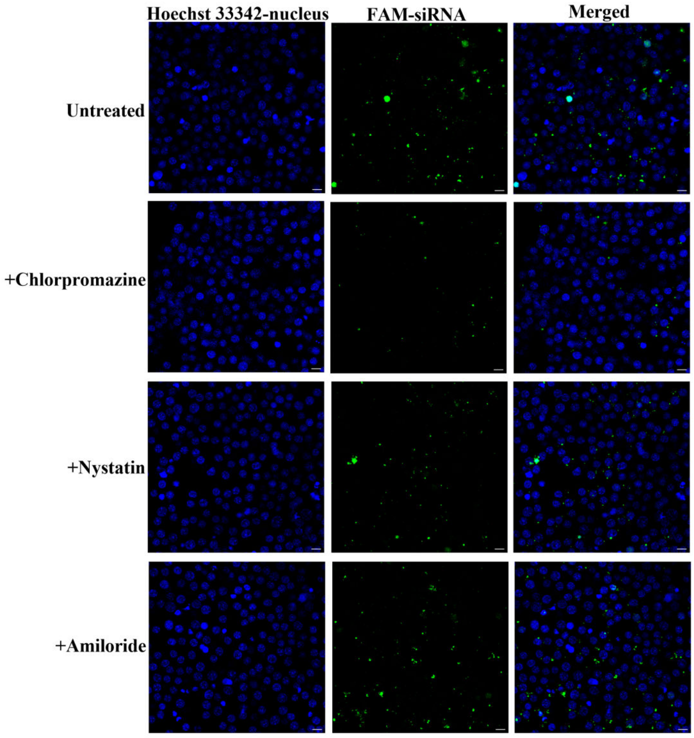

Fig. S11. Visualizing the endocytic mechanism of HVs@HA-BR-KDEL delivery. CLSM images of cellular uptake of HVs@HA-BR-KDEL after 12 h of incubation, with pretreatment of various endocytic inhibitors for 1 h at 37°C. Scale bar: 10 μm.

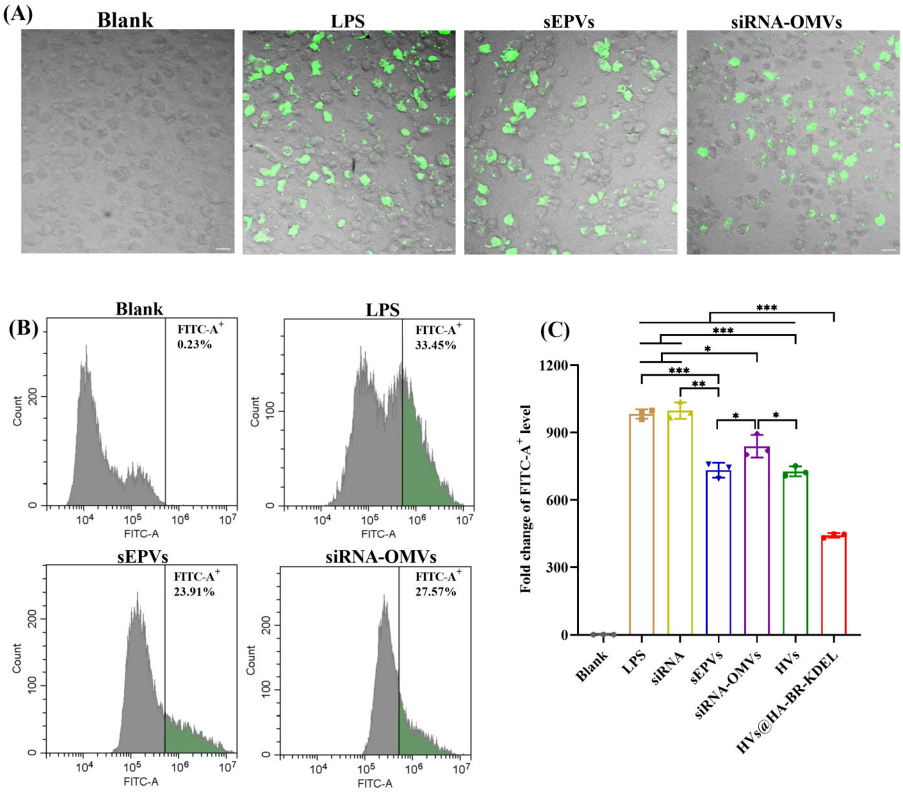

Fig. S12. Antioxidant effect of sEPVs and siRNA-OMVs. (A-C) sEPVs and siRNA-OMVs were evaluated for their ability to scavenge ROS in inflammatory RAW264.7 cells, as assessed by (A) CLSM, (B) flow cytometry, and (C) quantitative analysis of DCFH-DA fluorescence (n=3). Scale bar: 20 μm. Data are presented as the means ± SD. **p* < 0.05, ***p* < 0.01, ****p* < 0.001.

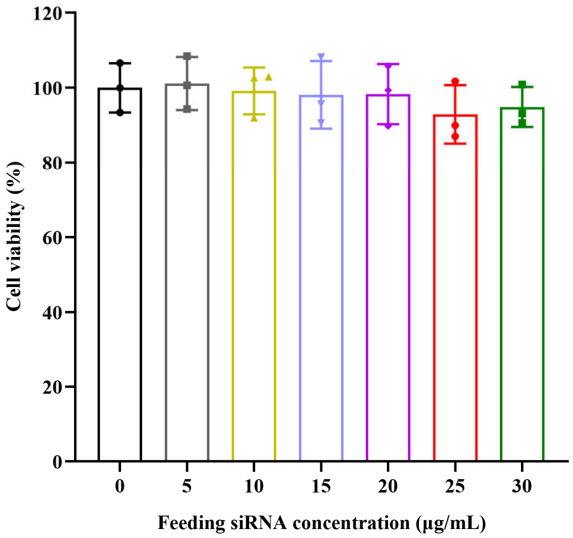

Fig. S13. Cytotoxicity assessment of the nanovesicles. Cell viability of RAW264.7 cells after treatment with HVs@HA-BR-KDEL at increasing initial feeding concentrations (5-30 μg/mL, siRNA equivalent) (n=3). Data are presented as the means ± SD. *p* < 0.05, ***p* < 0.01, ****p* < 0.001.

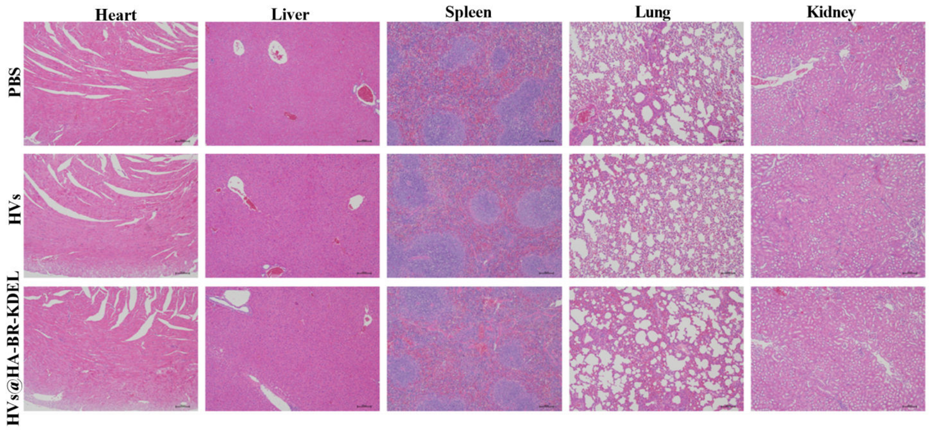

Fig. S14. ***In vivo*** **biosafety assessment of oral nanovesicles.** H&E staining images of major organs (heart, liver, spleen, lung, and kidney) from mice following oral gavage of nanovesicles for 4 days. Scale bar: 100 μm.

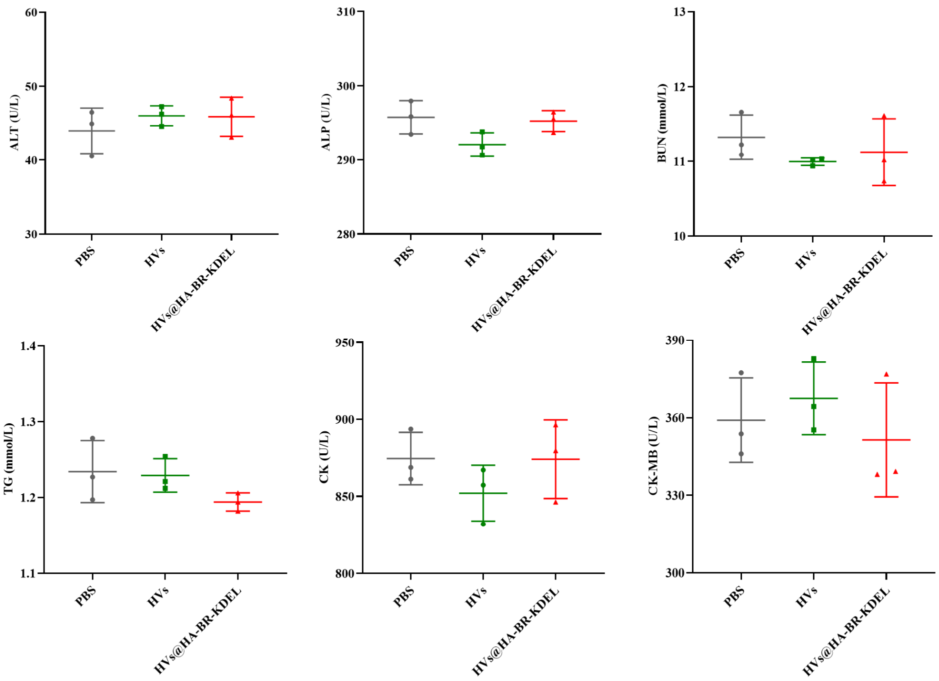

Fig. S15. Assessment of systemic safety by hematological analysis. Analysis of blood from mice after 4 days of oral gavage with nanovesicles (n=3). Major blood routine indices include the concentrations of alanine aminotransferase (ALT), alkaline phosphatase (ALP), blood urea nitrogen (BUN), triglyceride (TG), creatine kinase (CK), and creatine kinase-myocardial band isoenzyme (CK-MB). Data are presented as the means ± SD. *$p < 0.05$, **$p < 0.01$, ***$p < 0.001$.

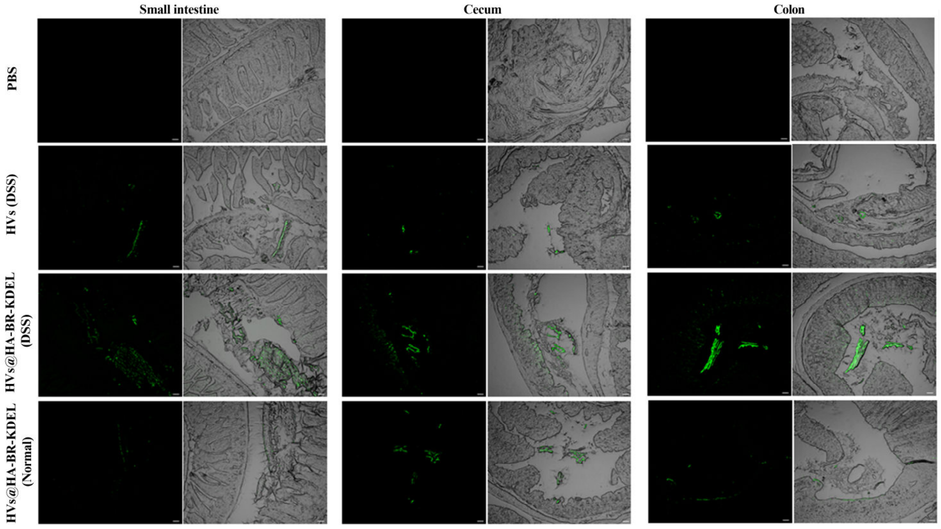

Fig. S16. Tracking the oral delivery of siRNA to the intestinal tract. Fluorescence images of the small intestine, cecum, and colon from mice after oral administration with nanovesicles for 24 h. Green: FAM-siRNA. Scale bar: 50 μm.

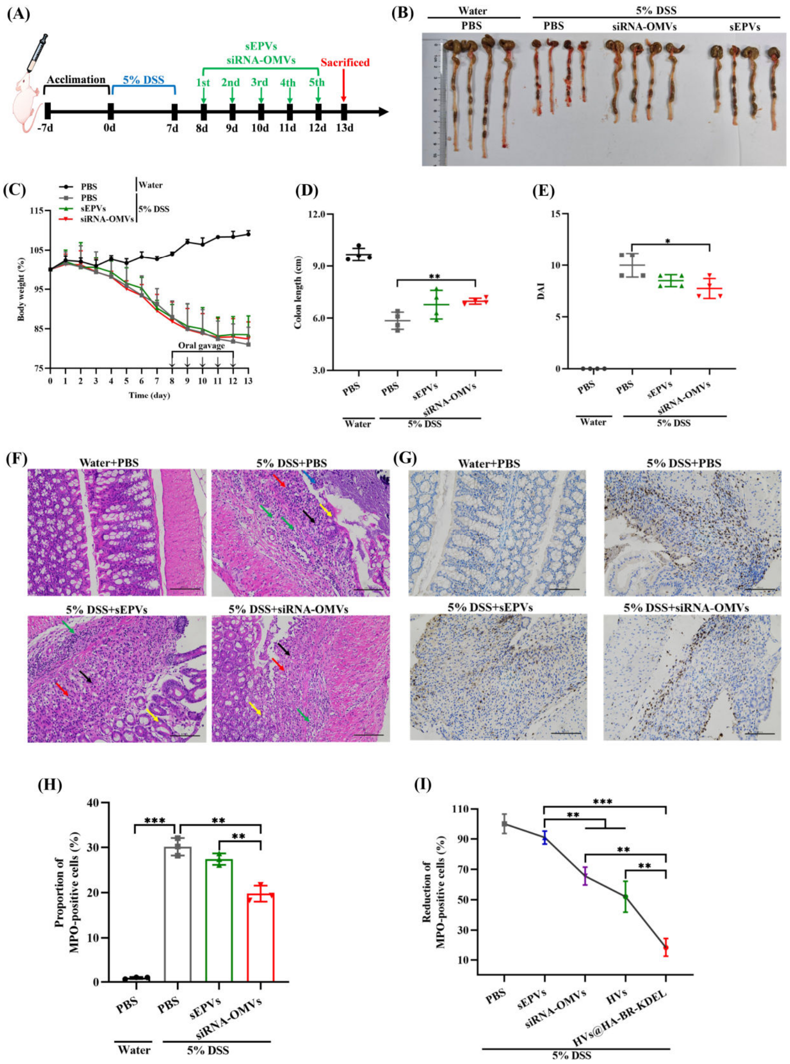

Fig. S17. Therapeutic efficacy of orally administered sEPVs and siRNA-OMVs in murine colitis. **(A)** Schematic illustration of experimental design for treatment. **(B)** Representative macroscopic images of harvested colons (n=4). **(C)** Daily body weight change of mice for 13 days (n=4). **(D)** Quantified lengths of colons (n=4). **(E)** DAI of mice at the end of the therapeutic regimen (n=4). **(F)** Representative H&E staining images of colon tissues. Scale bar: 100 μm. **(G)** Representative MPO staining images of colon tissues. Scale bar: 100 μm. **(H)** Quantification of MPO-positive cell proportion in colon tissues (n=3). **(I)** Reduction of MPO-positive cells in colon tissues after different treatments in colitis mice (n=3). Data are presented as the means ± SD. *$p < 0.05$, **$p < 0.01$, ***$p < 0.001$.

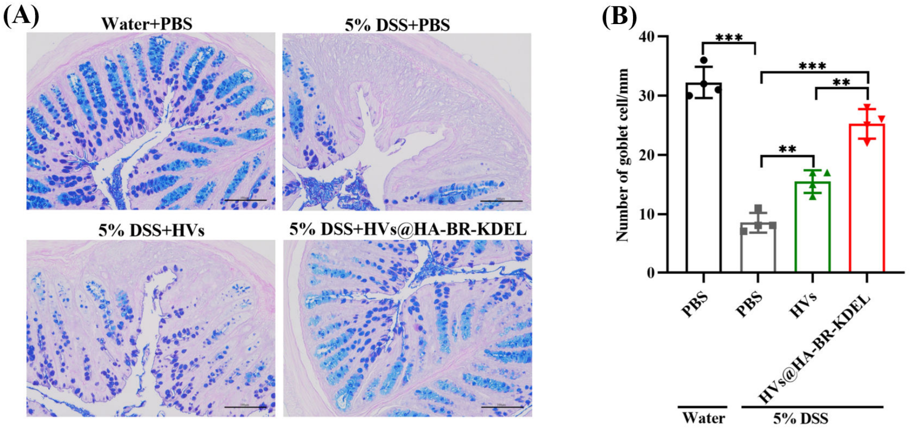

Fig. S18. AB/PAS staining of colon tissues. (A) Representative images. Scale bar: 100 μm. **(B)** Quantification of goblet cells per mm (n=4). Data are presented as the means ± SD. **p* < 0.05, ***p* < 0.01, ****p* < 0.001.

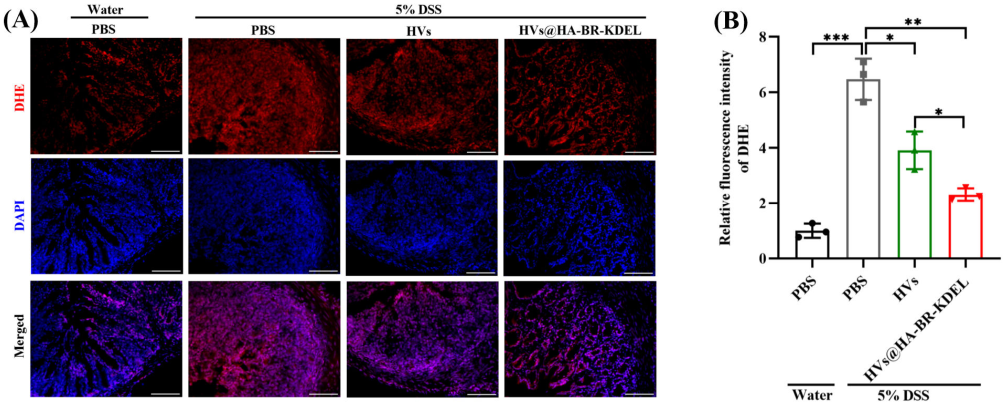

Fig. S19. ROS levels detected in colon tissues using DHE staining after HVs and HVs@HA-BR-KDEL treatments. (A) Representative fluorescence images. The nuclei were stained with DAPI (blue). Scale bar: 50 μm. **(B)** Corresponding quantitative analysis of fluorescence intensity (n=3). Data are presented as the means ± SD. **p* < 0.05, ***p* < 0.01, ****p* < 0.001.

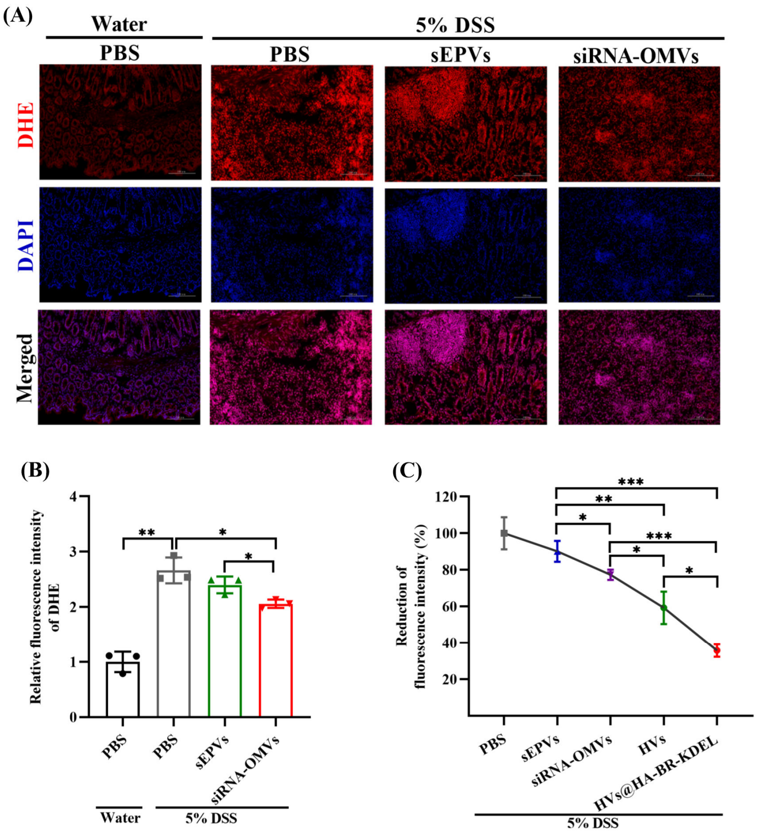

Fig. S20. ROS levels detected in colon tissues using DHE staining after sEPVs and siRNA-OMVs treatments. (A) Representative fluorescence images. The nuclei were stained with DAPI (blue). Scale bar: 100 μm. **(B)** Corresponding quantitative analysis of fluorescence intensity (n=3). **(C)** Reduction of fluorescence intensity of ROS after different treatments in colitis mice (n=3). Data are presented as the means ± SD. $*p < 0.05$, $**p < 0.01$, $***p < 0.001$.

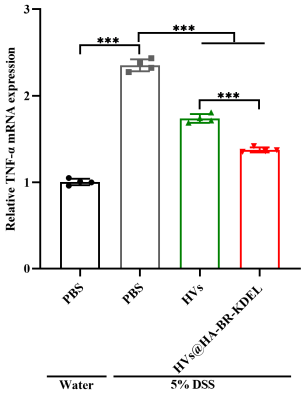

Fig. S21. Regulation of TNF-α gene expression in colon tissues from HVs and HVs@HA-BR-KDEL groups. The mRNA expression levels of TNF-α in colon tissues detected by RT-qPCR assays (n=4). Data are presented as the means ± SD. $*p < 0.05$, $**p < 0.01$, $***p < 0.001$.

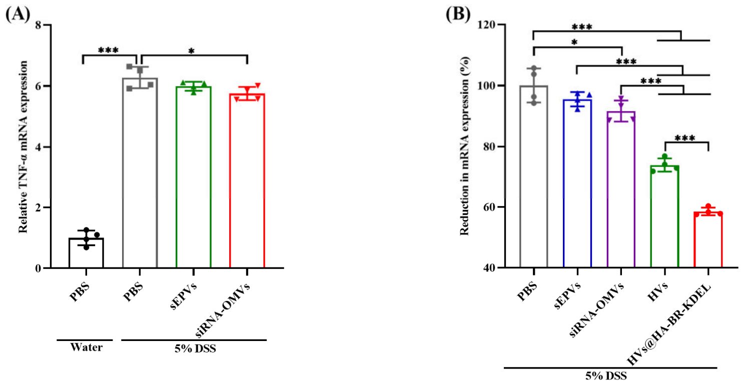

Fig. S22. Regulation of TNF-α gene expression in colon tissues from sEPVs and siRNA-OMVs groups. (A) The mRNA expression levels of TNF-α in colon tissues detected by RT-qPCR assays (n=4). **(B)** Reduction in mRNA expression after different treatments in colitis mice (n=4). Data are presented as the means ± SD. *$p < 0.05$, **$p < 0.01$, ***$p < 0.001$.

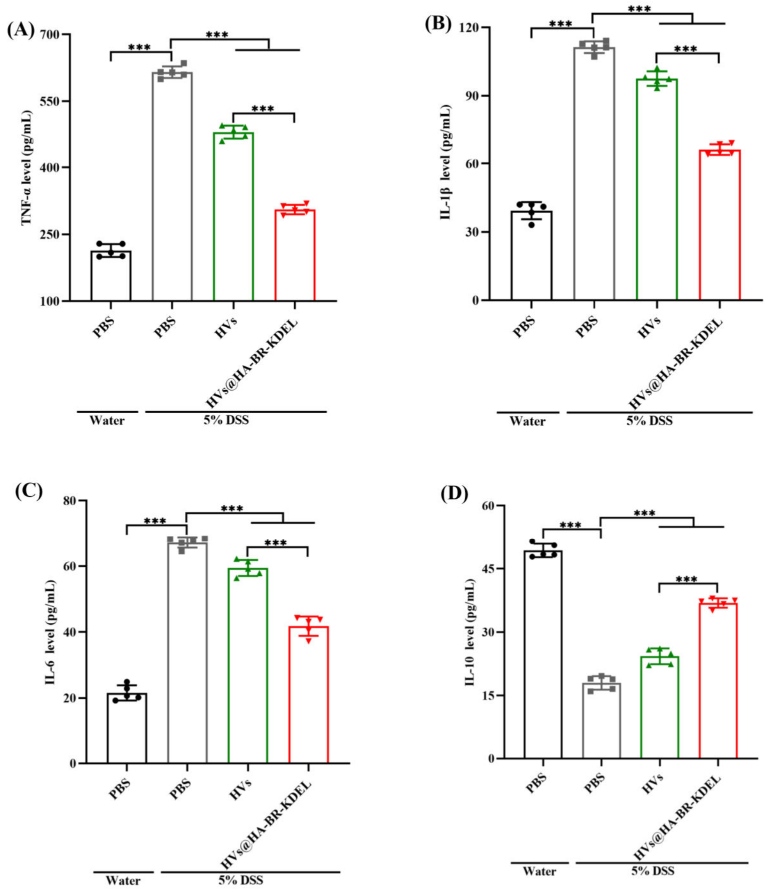

Fig. S23. Cytokine levels of serum samples from mice in different groups measured by ELISA assays. (A-D) Concentrations of (A)TNF-α, (B) IL-1β, (C) IL-6, and (D) IL-10 in serum samples (n=5). Data are presented as the means ± SD. *$p < 0.05$, **$p < 0.01$, ***$p < 0.001$.

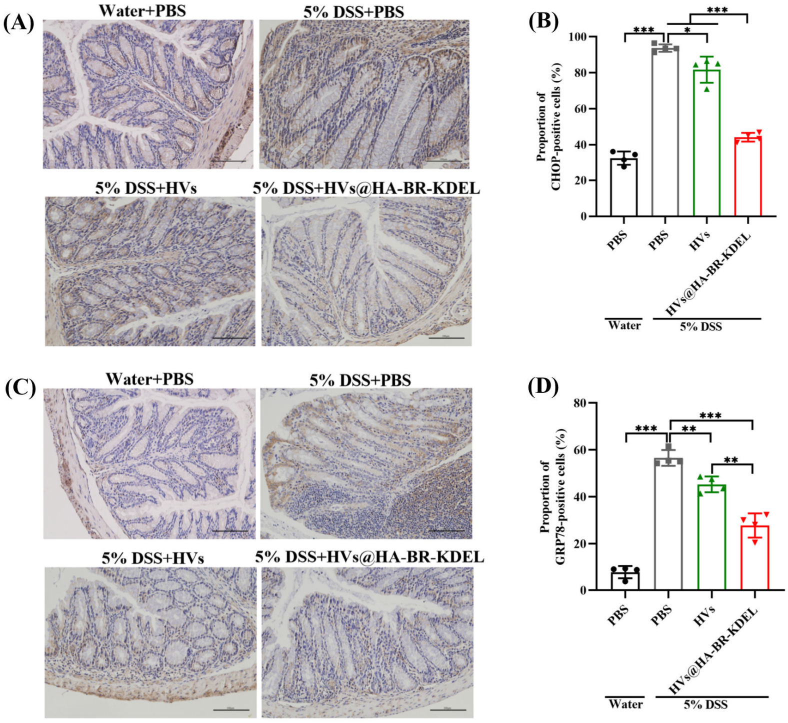

Fig. S24. Immunohistochemical staining for CHOP and GRP78 in colon tissues from different treatment groups. (A) and **(C)** Representative immunohistochemical images of (A) CHOP and (C) GRP78 expression. Scale bar: 100 µm. **(B)** and **(D)** Corresponding quantitative analysis of the proportion of (B) CHOP- and (D) GRP78-postive cells (n=4). Data are presented as the means ± SD. *$p < 0.05$, **$p < 0.01$, ***$p < 0.001$.

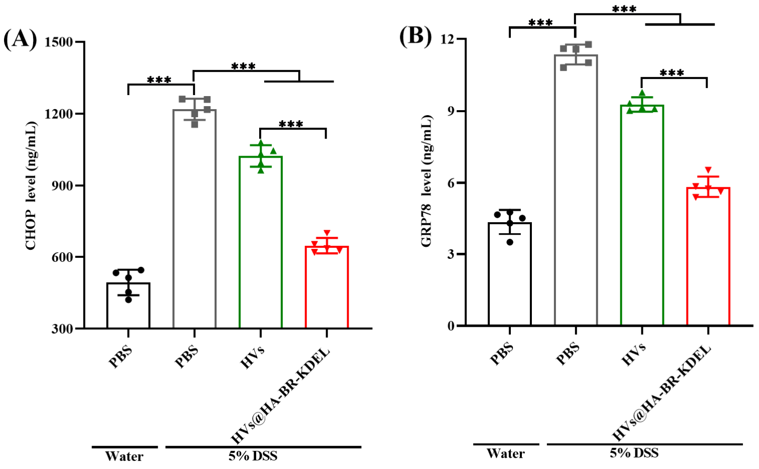

Fig. S25. Quantitative analysis of the expression levels of CHOP and GRP78 in colon tissues from each group. (A) and **(B)** Concentrations of (A) CHOP and (B) GRP78 in the colon tissues measured by ELISA assays (n=5). Data are presented as the means ± SD. *$p < 0.05$, **$p < 0.01$, ***$p < 0.001$.

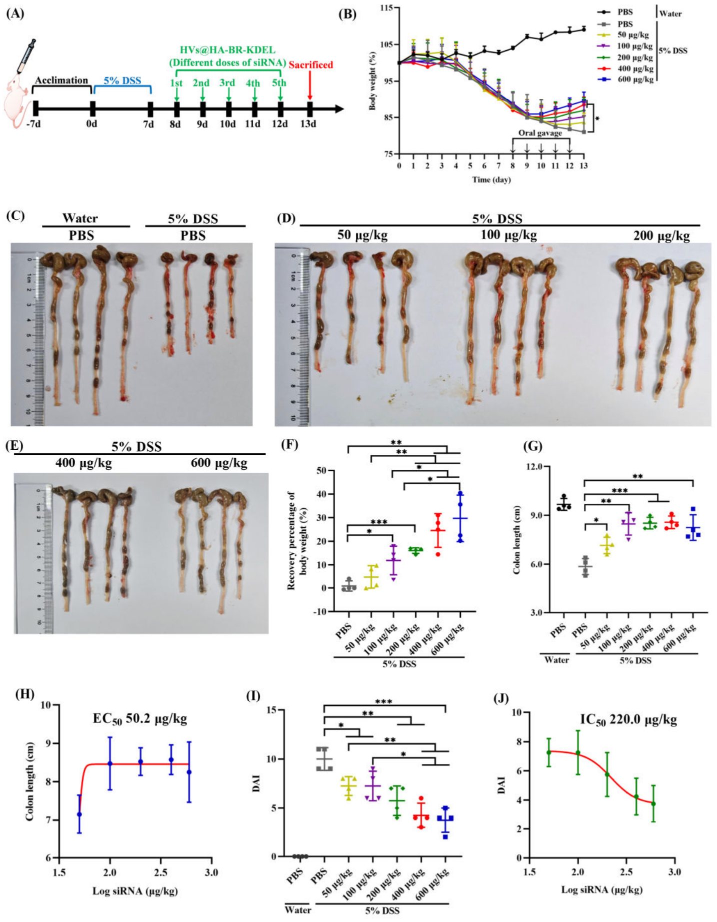

Fig. S26. Dose-ranging study of siRNA delivered by HVs@HA-BR-KDEL in murine colitis. (A) Schematic of the experimental timeline and dosing regimen. **(B)** Daily body weight change of mice for 13 days (n=4). **(C-E)** Representative macroscopic images of harvested colons from each dose group. **(F)** Dose-response analysis of final body weight recovery (n=4). **(G)** Quantification of colon lengths from each treatment group (n=4). **(H)** Dose-response curve of colon lengths, plotted against the logarithm of siRNA dose (μg/kg) (n=4). **(I)** DAI assessed at the study endpoint (day 13) (n=4). **(J)** Dose-response curve for DAI scores, plotted against the logarithm of siRNA dose (μg/kg) (n=4). Data are presented as the means ± SD. *$p < 0.05$, **$p < 0.01$, ***$p < 0.001$.

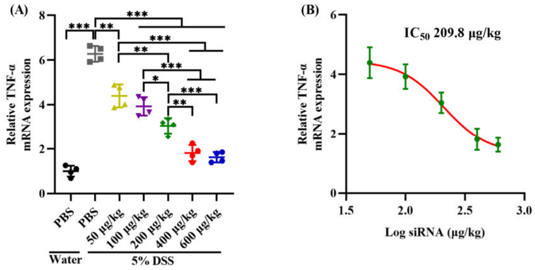

Fig. S27. Dose-dependent silencing of target gene expression in colon tissues. (A)

The mRNA expression levels of TNF-α detected by RT-qPCR assays (n=4). **(B)** Dose-response curve for mRNA expression levels, plotted against the logarithm of siRNA dose (μg/kg) (n=4). Data are presented as the means ± SD. *$p < 0.05$, **$p < 0.01$, ***$p < 0.001$.

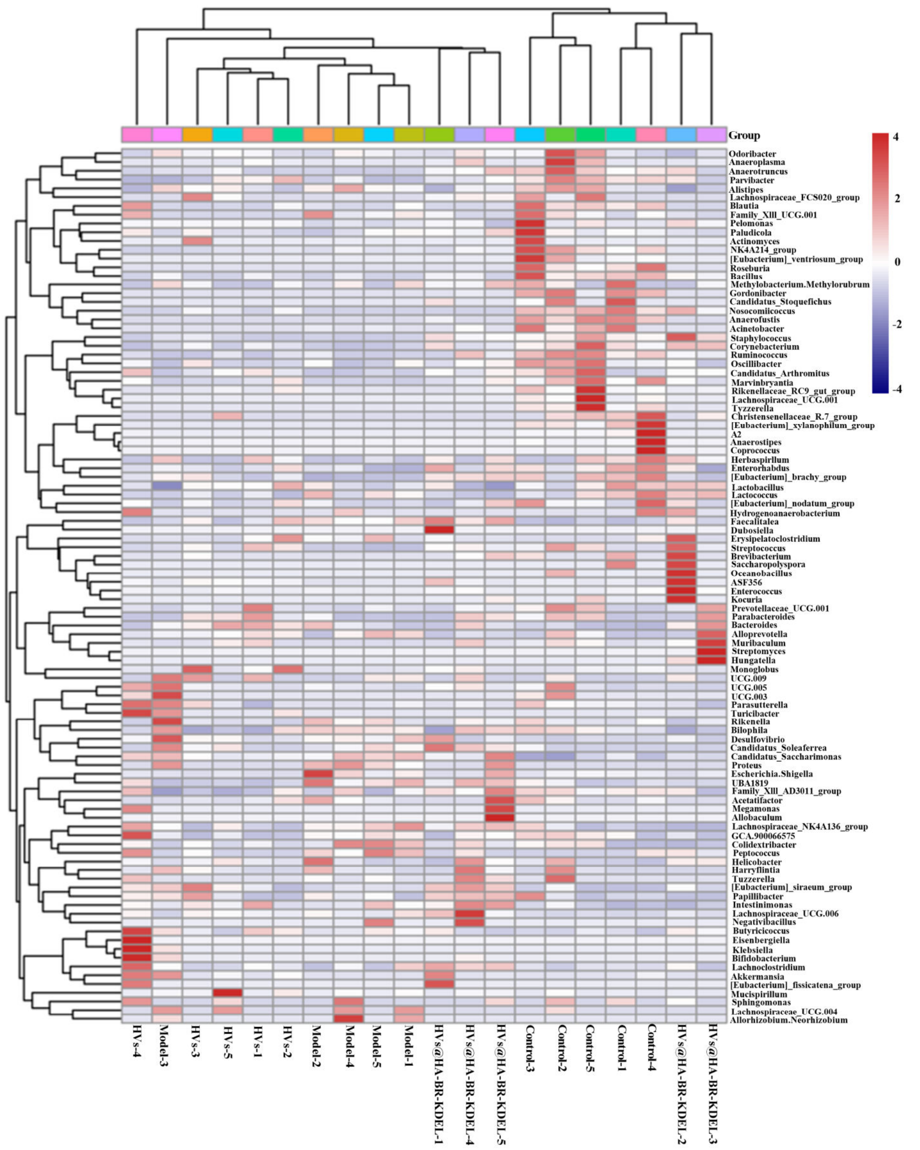

Fig. S28. Alterations in gut microbial community structure. Heatmap illustrating the distribution of gut microbiota at the genus level across different experimental groups.

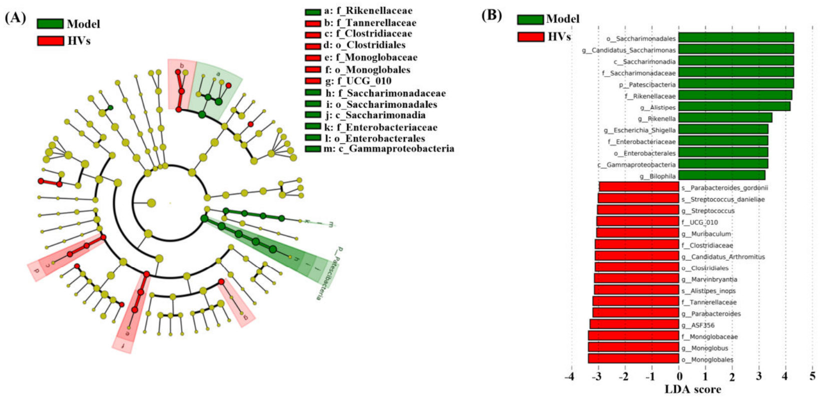

Fig. S29. LEfSe analysis of HVs intervention on gut microbiota of colitis mice. (A) LEfSe cladogram illustrating the differentially abundant bacterial lineages across groups. **(B)** Histogram of LDA scores for biomarkers with an LDA score greater than 2. The bar length corresponds to the effect size of each differentially abundant taxon.
